# Nonparametric IPSS: fast, flexible feature selection with false discovery control

**DOI:** 10.1093/bioinformatics/btaf299

**Published:** 2025-05-13

**Authors:** Omar Melikechi, David B Dunson, Jeffrey W Miller

**Affiliations:** Department of Biostatistics, Harvard T.H. Chan School of Public Health, Boston, MA, 02115, United States; Department of Statistical Science, Duke University, Durham, NC, 27708, United States; Department of Biostatistics, Harvard T.H. Chan School of Public Health, Boston, MA, 02115, United States

## Abstract

**Motivation:**

Feature selection is a critical task in machine learning and statistics. However, existing feature selection methods either (i) rely on parametric methods such as linear or generalized linear models, (ii) lack theoretical false discovery control, or (iii) identify few true positives.

**Results:**

We introduce a general feature selection method with finite-sample false discovery control based on applying integrated path stability selection (IPSS) to arbitrary feature importance scores. The method is nonparametric whenever the importance scores are nonparametric, and it estimates *q*-values, which are better suited to high-dimensional data than *P*-values. We focus on two special cases using importance scores from gradient boosting (IPSSGB) and random forests (IPSSRF). Extensive nonlinear simulations with RNA sequencing data show that both methods accurately control the false discovery rate and detect more true positives than existing methods. Both methods are also efficient, running in under 20 s when there are 500 samples and 5000 features. We apply IPSSGB and IPSSRF to detect microRNAs and genes related to cancer, finding that they yield better predictions with fewer features than existing approaches.

**Availability and implementation:**

All code and data used in this work are available on GitHub (https://github.com/omelikechi/ipss_bioinformatics) and permanently archived on Zenodo (https://doi.org/10.5281/zenodo.15335289). A Python package for implementing IPSS is available on GitHub (https://github.com/omelikechi/ipss) and PyPI (https://pypi.org/project/ipss/). An R implementation of IPSS is also available on GitHub (https://github.com/omelikechi/ipssR).

## 1 Introduction

Identifying the important features in a dataset can greatly improve performance and interpretability in machine learning and statistical problems (Theng and Bhoyar 2024). For example, in genomics, often only a small fraction of genes (features) are related to a disease of interest (response). By identifying these genes, scientists can save time and resources while gaining insights that would be difficult to uncover otherwise (Theng and Bhoyar 2024).

The goal of feature selection is to maximize the number of important features selected (true positives)—informally referred to here as *power*—while minimizing the number of unimportant features selected (false positives). In simulations, we find that popular methods without theoretical false discovery control, such as recursive feature elimination and Boruta (Kursa *et al.* 2010), often select many false positives (Section 3). Meanwhile, popular methods with false discovery control, namely stability selection (Meinshausen and Bühlmann 2010, Shah and Samworth 2013) and model-X knockoffs (Candes *et al.* 2018), have low power in simulations and select few features in practice (Sections 3 and 4).

One reason for stability selection’s low power is its relatively weak theoretical upper bounds on the expected number of false positives, E(FP). Recently, Melikechi and Miller (2024) proved that much stronger bounds hold for *integrated path stability selection* (IPSS), which consequently identifies more true positives. Until now, IPSS has only been applied to generalized linear models, limiting its applicability since parametric assumptions are often violated or difficult to verify in practice.

Thus, there is a need for nonparametric feature selection methods with theoretical false discovery control and greater power. In this work, we address this need by applying IPSS to arbitrary feature importance scores. The result is a general feature selection method with tight upper bounds on E(FP) that are characterized by novel quantities called *efp scores*. In addition to controlling E(FP), efp scores approximately control the false discovery rate (FDR) and estimate *q*-values for each feature, which are more reliable than *P*-values in genomics and other high-dimensional settings (Storey and Tibshirani 2003).

Our proposed method is nonparametric whenever the feature importance scores come from nonparametric models. We develop two specific instances of this: IPSS for gradient boosting (IPSSGB) and IPSS for random forests (IPSSRF). Like knockoffs, neither IPSSGB nor IPSSRF assume a specific functional relationship between the response and the features. Unlike knockoffs, neither method requires knowledge of the joint distribution of the features.

In simulations, we find that IPSSGB and IPSSRF provide a better balance of FDR control and power than 12 other methods, and that IPSSGB performs best overall. In particular, both methods significantly outperform parametric versions of IPSS when the parametric assumptions are violated. In Section 4, we find that IPSSGB and IPSSRF successfully identify microRNAs and genes related to ovarian cancer and glioma, achieving better predictive performance than other feature selection methods while using fewer features. Both are also computationally efficient.

Finally, another important aspect of feature selection is stability—the consistent selection of features across similar settings. Stability improves reproducibility, which is critical in many applications (Li *et al.* 2022). As their names suggest, stability selection and IPSS are designed to produce stable results by repeatedly applying a baseline feature selection algorithm to random subsamples of the data (Section 2.2). Nogueira *et al.* (2018) show that stability selection can produce significantly more stable results than its baseline algorithm. In this work, we focus on false discovery control and power; further study of the stability of IPSS, stability selection, and other stability-inspired methods like StabML-RFE (Li *et al.* 2022) is left to future work.

The rest of this article is organized as follows. In Section 2, we introduce efp scores, review IPSS, and present its extension to feature importance scores. In Section 3, we present our simulation studies, and in Section 4, we analyze ovarian cancer and glioma data. We conclude in Section 5 with a discussion.

## 2 Materials and methods

In Section 2.1, we introduce efp scores. These quantities, assigned to each feature in the dataset, are used to perform feature selection with E(FP) control. In Section 2.2, we introduce IPSS, which is a general approach for constructing efp scores. In Section 2.3, we describe how IPSS can be applied to any feature importance score, focusing in particular on importance scores from tree-based methods such as gradient boosting and random forests.

*Notation*. Throughout this work, *n* and *p* are the number of samples and features, respectively, and $Z_{1:n}=(Z_{1},\ldots ,Z_{n})$ is a collection of independent and identically distributed (iid) random vectors $Z_{i}=(X_{i},Y_{i})$, where each $X_{i}\in R^{p}$ is a vector of features and $Y_{i}\in R$ is a response variable. Features are identified by their indices j∈{1,…,p}, 1 is the indicator function, that is, 1(A)=1 if *A* is true and 1(A)=0 otherwise, ⌊·⌋ is the floor function, and E and P are expectation and probability, respectively. The iid assumption is standard in the feature selection literature, and is assumed by stability selection, knockoffs, and IPSS. It is also a reasonable assumption in many applications involving tabular data, which is our focus in this work. For example, genomic data from unrelated individuals are widely treated as independent.

### 2.1 efp scores

Suppose S⊆{1,…,p} is an unknown subset of important features that we wish to estimate using $Z_{1:n}$. An *efp (expected false positive) score* is a function $\mathrm{ef}p_{Z_{1:n}}:\{1,\ldots ,p\}\to [0,\infty )$ that depends on $Z_{1:n}$ and satisfies the following:

$$\text{For}\;\text{all}\;t\geq 0,\;\text{if}\;\hat{S}(t)=\{j:\mathrm{ef}p_{Z_{1:n}}(j)\leq t\}\;\text{then}\;E(\text{FP}(t))\leq t,$$

where $E(\text{FP}(t))=E|\hat{S}(t)\cap S^{c}|$ is the expected number of false positives in $\hat{S}(t)$. That is, the estimator $\hat{S}(t)$ of *S* selects at most *t* false positives on average. A trivial example of an efp score is $\mathrm{ef}p_{Z_{1:n}}(j)=p$ for all *j*. This corresponds to selecting either no features or all features. Specifically, if t∈[0,p), then $\hat{S}(t)=\emptyset$ and E(FP(t))=0≤t, while if t∈[p,∞), then $\hat{S}(t)=\{1,\ldots ,p\}$ and E(FP(t))≤t, since the number of false positives is at most *p*.

The quality of an efp score is measured by the tightness of its bounds E(FP(t))≤t. Better efp scores have tighter bounds because tight bounds enable accurate false positive control via the parameter *t*. Accurate control in turn leads to more true positives in $\hat{S}(t)$ since weak bounds overestimate the number of false positives, reducing the total number of features selected.

E(FP) and efp scores are related to two other quantities of significant interest: the false discovery rate (FDR) and *q*-values. Informally, the *false discovery rate* is the expected ratio between the number of false positives and the total number of features selected, FDR=E(FP/(TP+FP)), and the *q-value of feature j* is the smallest FDR when *j* is selected (Storey 2003). When *p* is large, as is often the case in genomics, we have

(2.1) $$\text{pFDR}(t)\approx \text{FDR}(t)\approx \frac{E(\text{FP}(t))}{E|\hat{S}(t)|}\leq \frac{t}{E|\hat{S}(t)|},$$

where $\text{pFDR}(t)=E(\text{FP}(t)/|\hat{S}(t)|||\hat{S}(t)|>0)$ is the *positive false discovery rate*, the two approximations are from Storey (2003), and the inequality holds by the definition of an efp score. It follows that the *q*-value of feature *j* satisfies

(2.2) $$q_{j}=\underset{\left\{t:\mathrm{efp}p(j)\leq t\right\}}{\inf}\text{pFDR}(t)≲\underset{\left\{t:\mathrm{efp}(j)\leq t\right\}}{\inf}\frac{t}{E|\hat{S}(t)|},$$

where the equality is the definition of the *q*-value (here, efp(j) denotes the observed value of the test statistic $\mathrm{ef}p_{Z_{1:n}}(j)$) (Storey 2003), and the approximate inequality ≲ holds by Equation (2.1). Thus, when the efp score has tight bounds, the *q*-value of feature *j* is well-approximated by the rightmost term in Equation (2.2), which is easily estimated in practice by replacing $E|\hat{S}(t)|$ with $\left|\hat{S}(t)\right|$. Similarly, by Equation (2.1), FDR(t) is approximately bounded by $t/|\hat{S}(t)|$. So, as an alternative to specifying the target E(FP) parameter *t*, one can control the FDR at level α by choosing the largest set $\hat{S}(t)$ such that $t/|\hat{S}(t)|\leq \alpha$. The largest such set is chosen to maximize true positives.

### 2.2 Integrated path stability selection

Integrated path stability selection (IPSS) constructs efp scores by applying baseline feature selection algorithms to random subsamples of the data. Specifically, let *S* be an unknown subset of true features as before, and let ${\hat{S}}_{\lambda }\subseteq \{1,\ldots ,p\}$ be an estimator of *S* that depends on the data and a parameter λ>0. Note that ${\hat{S}}_{\lambda }$ and $\hat{S}(t)$ are distinct estimators of *S*: The former is a baseline algorithm whose parameter λ will always appear as a subscript.

The IPSS subsampling procedure, which is also used in stability selection, consists of *B* subsampling iterations. Each iteration consists of randomly drawing disjoint subsets $A_{1},A_{2}\subseteq \{1,\ldots ,n\}$ of size ⌊n/2⌋ and evaluating ${\hat{S}}_{\lambda }(Z_{A_{1}})$ and ${\hat{S}}_{\lambda }(Z_{A_{2}})$ at all λ in some interval Λ⊆(0,∞), where $Z_{A}=(Z_{i}:i\in A)$ (the choice of ⌊n/2⌋ samples is needed for existing stability selection theorems—not just IPSS—to hold). After *B* iterations, the *estimated selection probability* ${\hat{\pi }}_{j}(\lambda )=\frac{1}{2B}\sum_{b=1}^{2B}1(j\in {\hat{S}}_{\lambda }(Z_{A_{b}}))$ of feature *j* is the proportion of times *j* is selected over all 2*B* subsets. Large values of ${\hat{\pi }}_{j}(\lambda )$ correspond to *j* being selected by ${\hat{S}}_{\lambda }$ on many of the random subsamples, suggesting that *j* is important.

Melikechi and Miller (2024) prove that for any Λ⊆(0,∞), any probability measure μ on Λ, and certain functions f:[0,1]→R, the function $\mathrm{ef}p_{Z_{1:n}}:\{1,\ldots ,p\}\to [0,p]$ defined by

(2.3) $$\mathrm{ef}p_{Z_{1:n}}(j)=\min\left\{\frac{I(\Lambda )}{\int_{\Lambda }f({\hat{\pi }}_{j}(\lambda ))\mu (d\lambda )},\;p\right\},$$

is a valid efp score, that is, $\hat{S}(t)=\{j:\mathrm{ef}p_{Z_{1:n}}(j)\leq t\}$ satisfies $E|\hat{S}(t)\cap S^{c}|\leq t$. An explicit form of I(Λ), which comes from the theory of IPSS, is provided in Section 1, available as supplementary data at Bioinformatics online.

Several choices of *f* in Equation (2.3) yield provably valid efp scores (see Section 1, available as supplementary data at Bioinformatics online as well as Theorems 4.1 and 4.2, Theorems 3.2 and 3.3, available as supplementary data at Bioinformatics online in Melikechi and Miller (2024) for theoretical results about IPSS with different functions). We always use $f(x)={\left(2x-1\right)}^{3}1(x\geq 0.5)$ because the resulting bounds E(FP(t))≤t are the tightest of any existing version of stability selection (Melikechi and Miller 2024). In addition to its theoretical justification, the empirical results in Figs 18 and 19, available as supplementary data at Bioinformatics online show that this choice of *f* produces the best results among several functions for which valid efp scores are available. Further details about IPSS, including descriptions of Λ, μ, and a derivation of Equation (2.3), are provided in Section 1, available as supplementary data at Bioinformatics online.

### 2.3 IPSS for feature importance scores

Until now, IPSS has only been applied to parametric baseline estimators ${\hat{S}}_{\lambda }$. A canonical example is $\ell^{1}$-regularized estimators ${\hat{S}}_{\lambda }=\{j:{\hat{\beta }}_{j}(\lambda )\neq 0\}$, where

(2.4) $$\hat{\beta }(\lambda )=\underset{\beta \in R^{p}}{\text{arg min}}\sum_{i=1}^{n}L(Y_{i},\beta^{T}X_{i})+\lambda \sum_{j=1}^{p}|\beta_{j}|.$$

Here, λ>0 controls the strength of the $\ell^{1}$-penalty and L is a log-likelihood that assumes a specific relationship between the features and response. By extending IPSS to feature importance scores, we no longer require such restrictive assumptions.

An *importance function* is a (possibly random) map $\Phi_{Z_{1:n}}:\{1,\ldots ,p\}\to R$ that uses the data to assign an *importance score* $\Phi_{Z_{1:n}}(j)$ to each feature. The possible randomness, which is additional to the randomness in $Z_{1:n}$, can come from, for example, random subsampling in tree-based algorithms. Suppressing $Z_{1:n}$ from the notation for now, assume Φ(j)<Φ(k) means that *j* is less important than *k* according to Φ. For example, in linear regression where $Z_{i}=(X_{i},Y_{i})$ with $Y_{i}=\beta^{T}X_{i}+\epsilon_{i}$ and $\epsilon_{i}∼N(0,\sigma^{2})$, a viable importance function is the magnitude of the estimated regression coefficient, $\Phi (j)=|{\hat{\beta }}_{j}|$.

Associated to any importance function Φ is a baseline feature selection estimator ${\hat{S}}_{\lambda }=\{j:\Phi (j)\geq \lambda \}$, obtained by simply thresholding the importance scores. That is, given Φ and a threshold λ, ${\hat{S}}_{\lambda }$ selects the features whose importance scores are at least λ. With ${\hat{S}}_{\lambda }$ defined, we have all that is needed to implement IPSS. Furthermore, all of the theoretical results from Melikechi and Miller (2024) apply without modification.

#### 2.3.1 Implementation

Algorithm 1, available as supplementary data at *Bioinformatics* online outlines IPSS for feature importance. The main steps are as follows: First, features are preselected according to the procedure described in Section 2.1, available as supplementary data at *Bioinformatics* online. This is a common preliminary step in many feature selection algorithms. Next, importance scores are evaluated for the preselected features using random, disjoint halves of the data. This process is repeated *B* times, yielding 2*B* importance scores for each feature. We then construct a grid of λ values. The largest, $\lambda_{\text{max}}$, is the maximum importance score across all features and all 2*B* sets of scores (hence, ${\hat{S}}_{\lambda_{\text{max}}}=\emptyset$). Next, starting from $\lambda_{\text{min}}=\lambda_{\text{max}}$, decrease $\lambda_{\text{min}}$ one step at a time, usually on a log scale, iteratively updating the integral $I([\lambda_{\text{min}},\lambda_{\text{max}}])$ at each step in the form of a Riemann sum approximation until $I([\lambda_{\text{min}},\lambda_{\text{max}}])$ surpasses a cutoff *C*. Once *C* is surpassed, the while loop stops and the feature-specific selection probabilities and integrals are evaluated. The algorithm outputs efp scores for each feature, which are used to control E(FP), FDR, and compute *q*-values, as described in Section 2.1.

Sensitivity analyses in Section 5, available as supplementary data at *Bioinformatics* online show that IPSS depends little on *C* and the number of subsamples *B* (in related work, Shah and Samworth (2013) also report that stability selection is insensitive to *B*). Our defaults are C=0.05 and B=100. For μ, we consider the family of measures $\mu_{\delta }(d\lambda )=z_{\delta }^{-1}\lambda^{-\delta }d\lambda$, where δ∈R and $z_{\delta }=\int_{\Lambda }\lambda^{-\delta }d\lambda$ is a normalizing constant that is easily computed in closed form (Melikechi and Miller 2024). The values δ=0 and δ=1 correspond to averaging over Λ on linear and log scales, respectively. Like *C* and *B*, sensitivity analyses in Section 5, available as supplementary data at Bioinformatics online show that IPSS is robust to the choice of δ. In regression problems, we use δ=1.25 for IPSSGB and IPSSRF. In classification, we use δ=1 for IPSSGB and δ=1.25 for IPSSRF.

#### 2.3.2 IPSSGB and IPSSRF

Any importance function can be combined with IPSS, providing many possible directions for future work. In this article, we focus on importance functions from tree ensemble methods because they are nonparametric, computationally efficient, and produce state-of-the-art results on tabular data (Shwartz-Ziv and Armon 2022). These importance functions are defined as follows; for additional details, see Louppe *et al.* (2013) or Biau and Scornet (2016).

Given a collection of binary decision trees, T, define

(2.5) $$\Phi (j)=\frac{1}{\left|T\right|}\sum_{T\in T}\sum_{v\in T}\Delta \varphi (v)1(j=j_{v})$$

where the outer sum is over all trees T∈T, the inner sum is over all nodes v∈T, $j_{v}$ is the feature used to split node *v*, and φ(v) measures the impurity of *v*. The change in impurity

$$\Delta \varphi (v)=\varphi (v)-\left(\frac{\left|v_{L}\right|}{\left|v\right|}\varphi (v_{L})+\frac{\left|v_{R}\right|}{\left|v\right|}\varphi (v_{R})\right)$$

is the impurity difference between *v* and its children, $v_{L}$ and $v_{R}$. Large positive values of Δφ(v) indicate that the feature $j_{v}$ used to split node *v* successfully partitions the data in a manner that is consistent with the objective of the tree.

For regression, we use the *squared error loss* impurity function, $\varphi (v)=\sum_{i\in v}{\left(Y_{i}-{\bar{Y}}_{v}\right)}^{2}/|v|$, where v⊆{1,…,n} is identified with the subset of samples in node *v*, and ${\bar{Y}}_{v}=\sum_{i\in v}Y_{i}/|v|$ is the empirical mean of the responses in *v*. For binary classification, we use the *Gini index*, $\varphi (v)=2p_{0}(v)p_{1}(v)$ where, for a∈{0,1}, $p_{a}(v)=\sum_{i\in v}1(Y_{i}=a)/|v|$ is the proportion of responses in *v* that equal *a*. These were selected because they are canonical choices and because we found little difference in results between these and other standard impurity functions.

The importance function defined in Equation (2.5) can be computed for any tree ensemble method. We focus on gradient boosting (Friedman 2001) and random forests (Breiman 2001). As noted above, these are abbreviated IPSSGB and IPSSRF when combined with IPSS. Additional implementation details about IPSSGB and IPSSRF are in Sections 2.2.1 and 2.2.2, available as supplementary data at Bioinformatics online, respectively.

#### 2.3.3 Other importance functions

The importance function in Equation (2.5) is called *mean decrease impurity* (MDI) because it measures the average decrease in impurity attributed to each feature over all trees. Another common importance function is *mean decrease accuracy* (MDA), also known as *permutation importance* (Louppe *et al.* 2013).

MDA is more general than MDI in that it can be applied to any predictive model, not just tree ensembles. Several variants of MDA exist, but the basic procedure is: (i) train the model, (ii) compute the prediction error *e* on test data, and (iii) for each feature *j*, randomly permute the values of feature *j* in the test data (keeping all other features unchanged), and compute the prediction error $e_{j}$ of the trained model on the permuted test data. The underlying idea, captured by the importance score $\Phi (j)=e_{j}-e$, is that permuting unimportant features should have little effect on the prediction error, while permuting important features should degrade predictive performance.

We experimented with IPSS applied to both MDI and MDA from gradient boosting and random forests. In both cases, the FDR was controlled at target levels, but IPSS with MDI consistently identified more true positives. One likely reason for this is that MDA tends to spread importances more uniformly across features than MDI, making it more difficult to distinguish between important and unimportant features (Hastie *et al.* 2009). Furthermore, MDI, whose importance scores are obtained during the training process itself, is more computationally efficient than MDA, which requires the additional step of evaluating the trained model on the permuted data for every feature.

The recent success of deep learning, especially on text and image data, has generated much interest in feature importance scores—for example, SHAP values (Lundberg and Lee 2017)—that are derived from these models. Applying IPSS to such scores is a potentially interesting line of future work. However, numerous studies have shown that tree ensemble methods like XGBoost (Chen and Guestrin 2016) are faster, easier to train, and consistently outperform deep learning methods on tabular data, especially when there are fewer than 10 000 samples (Grinsztajn *et al.* 2022, Shwartz-Ziv and Armon 2022, Fayaz *et al.* 2022). Since our focus in this work is on tabular medical data, where the number of patients rarely exceeds several hundred, we primarily consider importance scores from tree ensemble methods given their many advantages over deep learning in this setting.

#### 2.3.4 Computation

The IPSS subsampling procedure requires 2*B* feature importance evaluations, one evaluation on each half of the data in all *B* iterations. This is fast for MDI scores; when combined with preselection (Section 2.1, available as supplementary data at Bioinformatics online), IPSSGB and IPSSRF run in under 20 s on a standard laptop when there are n=500 samples and P=5000 features (Tables 3 and 4, available as supplementary data at *Bioinformatics* online). Since this is already sufficiently fast for our purposes, we do not implement more efficient alternatives in this work.

We note, however, that IPSS is embarrassingly parallel: All iterations of the subsampling procedure can be evaluated separately and hence run simultaneously, potentially accelerating IPSS by a factor of 2*B* (typically 100 or 200). This is especially beneficial when importance scores are expensive to compute, as is often the case when they come from deep learning methods. By contrast, many feature selection methods are iterative and thus not parallelizable. For example, recursive feature elimination (Li *et al.* 2022) typically removes features one at a time based on predictive performance, requiring the model to be retrained after each removal. When *p* is large, this can require thousands of model fits, far more than the 2*B* evaluations typically required by IPSS.

## 3 Simulation studies

In this section, we conduct two simulation studies to evaluate the performance of 14 feature selection methods when the true set of important features is known. Features in the first study are drawn from a multivariate Gaussian, and the response is generated from a nonlinear additive model. To make the simulations more realistic, in the second study we use features from real RNA sequencing (RNA-seq) data rather than generating them from known distributions, and the response is a highly randomized, nonlinear function of the important features.

### 3.1 Other methods

We compare IPSSGB and IPSSRF to 12 feature selection methods: IPSS with $\ell^{1}$-regularization (IPSSL1, Melikechi and Miller 2024); boosting with stability selection (SSBoost, Hofner *et al.* 2015); five versions of model-X knockoffs (Candes *et al.* 2018), namely knockoffs with generalized linear models (KOGLM), knockoffs with lasso (KOL1), knockoffs with random forests (KORF), knockoffs with boosted trees (KOBT, Jiang *et al.* 2021), and knockoffs with deep neural networks (DeepPINK, Lu *et al.* 2018); random forest hypothesis testing (RFHT, Coleman *et al.* 2022); Boruta (Kursa *et al.* 2010); recursive feature elimination with gradient boosting (RFEGB); Vita (Janitza *et al.* 2018); and VSURF (Genuer *et al.* 2010). We provide a brief overview of these methods below. Additional details, including parameter settings and the software packages used to implement each method, are in Section 2, available as supplementary data at Bioinformatics online.

Four methods—Boruta, RFEGB, Vita, and VSURF—do not have theoretical false discovery control. We chose these because Speiser *et al.* (2019) found that Boruta and VSURF were among the best out of 13 random forest-based feature selection methods, and Degenhardt *et al.* (2019) found that Boruta and Vita outperformed 5 other methods in extensive comparison studies. We tested RFEGB so as to include a gradient boosting-based feature selection method without theoretical false discovery control.

IPSSL1 and SSBoost provide theoretical E(FP) control. IPSSL1, a parametric version of IPSS, assumes a (generalized) linear relationship between the features and the response (Equation 2.4). SSBoost combines gradient boosting and stability selection. We implement it assuming the *r*-concavity conditions of Shah and Samworth (2013), which are required to obtain the tightest upper bound on E(FP) of any version of stability selection other than IPSS (note that IPSS does not require *r*-concavity assumptions) (Melikechi and Miller 2024).

Model-X knockoffs is a general framework for feature selection with theoretical FDR control that has attracted much attention recently, in part due to its flexibility. Like IPSS, model-X knockoffs works in high-dimensions (p>n), does not use *P*-values (which are challenging to compute in general), is compatible with arbitrary feature importance scores, and makes no assumptions about the relationship between the response and the features. Unlike IPSS, model-X knockoffs assumes that the joint distribution of the features is known (Candes *et al.* 2018).

### 3.2 Gaussian simulation design

We perform two regression experiments and two classification experiments. The two experiments in both settings correspond to n=250 and 500. All experiments consist of 100 trials. In each trial, *n* independent samples of X∼N(0,Σ) are drawn from a p=500-dimensional, mean-zero multivariate Gaussian with a Toeplitz covariance matrix, Σ. The correlation parameter of the Toeplitz matrix is set to ρ=0.5; that is, $\Sigma_{jk}=0.5^{\left|j-k\right|}$.

The number of important features |S| is drawn uniformly at random from {5,…,15} prior to each trial, and a new set of important features *S* of size |S| is randomly selected. In each experiment, the signal is $f(X)=\sum_{j\in S}\;\text{exp}\;(-X_{j}^{2})$. For regression, the response is Y=f(X)+ϵ where $\epsilon ∼N(0,\sigma^{2})$ and $\sigma^{2}$ is selected according to a specified signal-to-noise ratio (SNR) that is drawn uniformly at random from the interval [0.5, 2]. For classification, we draw Y∼Bernoulli(π) where π=1/(1+exp(−uf(X))) and the signal strength *u* is drawn uniformly at random from the interval [1, 3]. These sources of randomness are introduced so that the study covers a wide range of settings.

### 3.3 RNA-seq simulation design

We perform three regression experiments and three classification experiments. The three experiments in both settings correspond to P=500, 2000, and 5000. All experiments consist of 100 trials. In each trial, n=500 patients and *P* genes are randomly selected from RNA-seq measurements of 6426 genes from 569 ovarian cancer patients (Vasaikar *et al.* 2018). This publicly available dataset, part of The Cancer Genome Atlas (Weinstein *et al.* 2013), was chosen because it is high dimensional and the features follow a variety of empirical distributions (Fig. 5, available as supplementary data at Bioinformatics online). Furthermore, the genes exhibit complex correlation structures, with maximum and average absolute pairwise correlations of approximately 0.95 and 0.17 after standardization, respectively.

Algorithm 2, available as supplementary data at *Bioinformatics* online describes the simulation procedure for each individual trial, which is also illustrated in Fig. 3, available as supplementary data at *Bioinformatics* online. In all steps, “randomly select” means select a parameter uniformly at random from its domain. The general outline is as follows: First, randomly select an *n*-by-*p* submatrix *X* of the full RNA-seq dataset and standardize its columns to have mean 0 and variance 1. Next, the number of important features |S| is drawn uniformly at random from {10,…,30} and a randomly selected subset of |S| important features *S* is partitioned into *G* groups, $S_{1},\ldots ,S_{G}$. A different realization of a randomized nonlinear function $f_{\theta_{g}}$, defined in Equation (3.1), available as supplementary data at Bioinformatics online, is applied to the standardized sum $\xi_{g}=({\hat{\xi }}_{g}-{\bar{\xi }}_{g})/{\bar{\sigma }}_{g}$ of the features in each group $S_{g}$, where ${\bar{\xi }}_{g}$ and ${\bar{\sigma }}_{g}$ are the empirical mean and standard deviation of ${\hat{\xi }}_{g}=\sum_{j\in S_{g}}X_{j}$. The resulting values are summed over all groups to generate a signal $\eta =\sum_{g=1}^{G}f_{\theta_{g}}(\xi_{g})$, and noise is added to this signal to generate a response *Y*. This scheme produces data with highly complex interactions between features and the response, going well beyond the additive setting $Y=\sum_{j\in S}f_{j}(X_{j})+\epsilon$.

For regression, the response is Y=η+ϵ, where $\epsilon ∼N(0,\sigma^{2})$ and the variance $\sigma^{2}$ is selected according to a specified signal-to-noise ratio (SNR) that is drawn uniformly at random from the interval [0.5, 2]. For classification, we draw Y∼Bernoulli(π) where π=1/(1+exp(−uη)) and the signal strength *u* is drawn uniformly at random from [1, 3]. As in the Gaussian simulation experiments, these many sources of randomness are introduced so that the simulations cover a wide range of settings.

### 3.4 Simulation results

**Figure 1.**
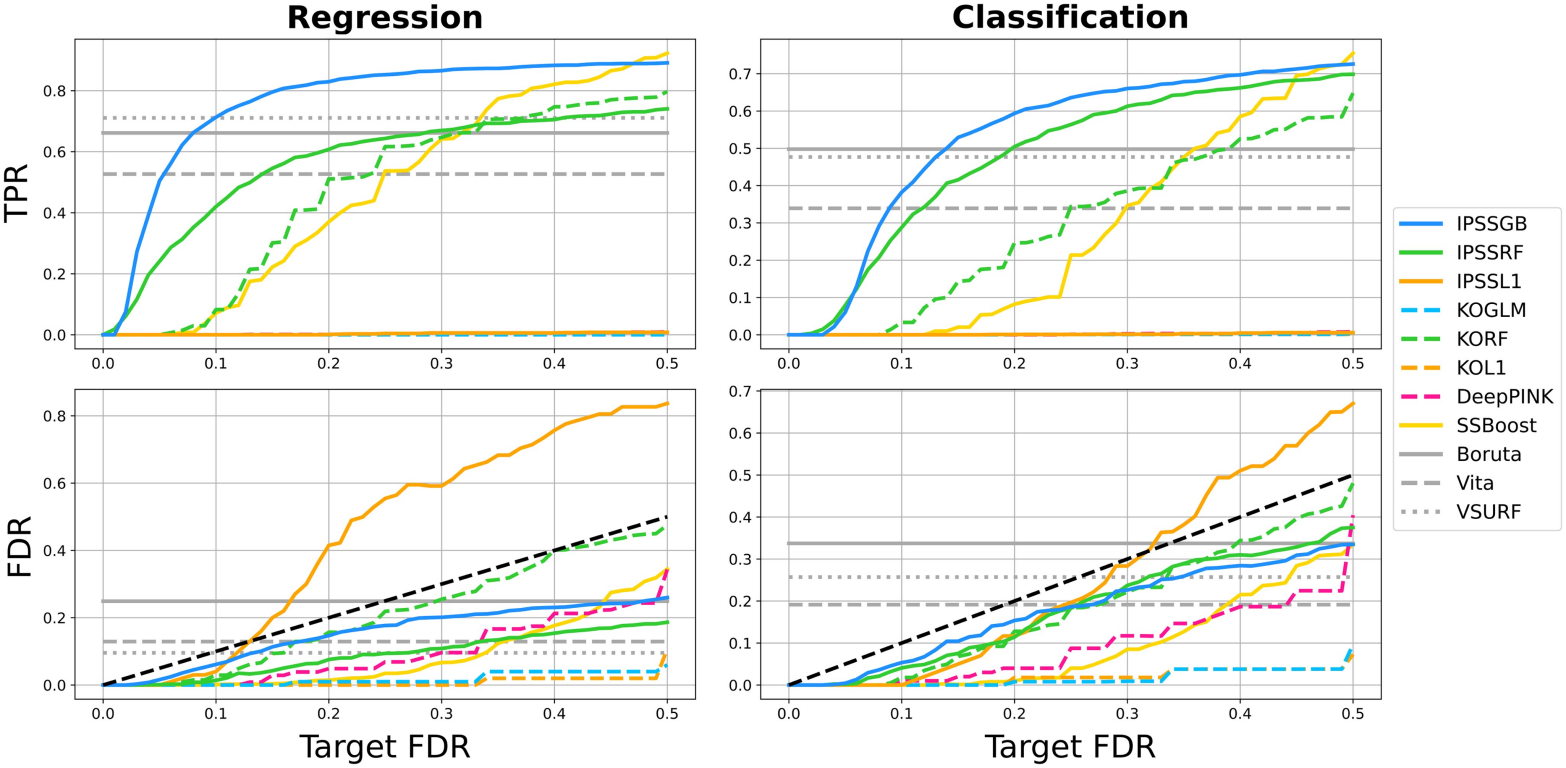
*Gaussian simulation results (* n=500 *)*. First and second columns show the regression and classification results, respectively. The horizontal axis in each plot shows the target FDR. The three methods without false discovery control (gray horizontal lines) do not vary with the target FDR. The black dashed line in the FDR plots represents perfect FDR calibration, FDR = target FDR. Non-gray solid lines correspond to IPSS or stability selection-based methods. Non-gray dashed lines show methods based on model-X knockoffs.

**Figure 2.**
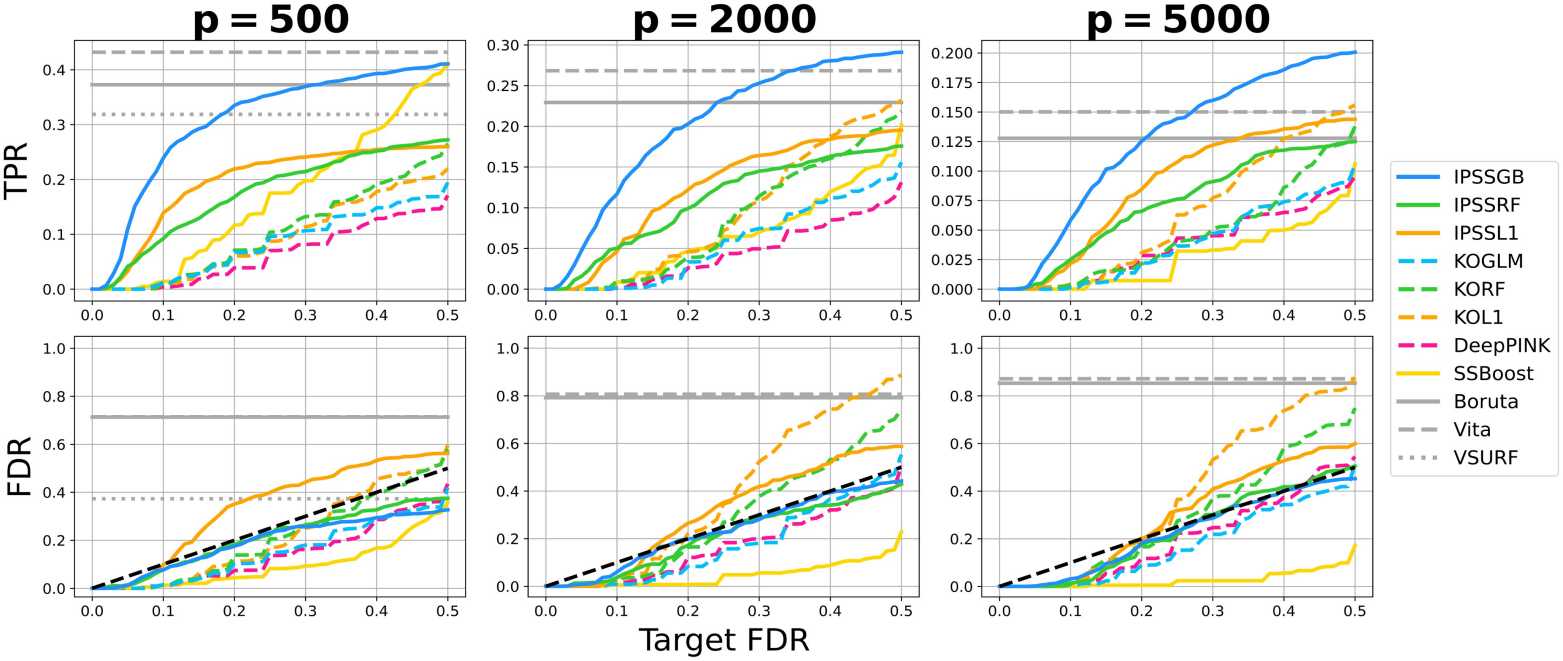
*RNA-seq simulation results (regression)*. First, second, and third columns correspond to the P=500, 2000, and 5000 experiments, respectively. The horizontal axis in each plot shows the target FDR. Methods without false discovery control (gray horizontal lines) do not vary with the target FDR. The black dashed line in the FDR plots represents perfect FDR calibration, FDR = target FDR. Non-gray solid lines correspond to IPSS or stability selection-based methods. Non-gray dashed lines show methods based on model-X knockoffs.

Figure 1 and Fig. 1, available as supplementary data at *Bioinformatics* online show the results of the Gaussian simulations when n=500 and 250, respectively, and Fig. 2 and Fig. 2, available as supplementary data at *Bioinformatics* online show the results of the RNA-seq simulations for regression and classification. Runtimes for each method in each experiment are provided in Tables 3 and 4, available as supplementary data at Bioinformatics online. The FDR in each plot is the average of FP/(TP+FP) over all 100 trials, and the true positive rate (TPR) is the average of TP/(TP+FN), where FP, TP, and FN are the number of false positives, true positives, and false negatives, respectively.

Both FDR and TPR are shown as functions of the target FDR. The black dashed line in each FDR plot represents perfect FDR calibration, FDR=target FDR. A method’s FDR is well-controlled if its FDR lies on or below this line. The FDR and TPR for methods without false discovery control are shown as horizontal lines because they do not admit false discovery control parameters and therefore do not vary with the target FDR.

IPSSGB has the best performance overall. Its FDR is always well-controlled and it consistently has a much higher TPR than the other methods with false discovery control. Notably, IPSSGB identifies significantly more true positives than other methods with false discovery control at lower target FDRs. For example, in the regression results in Fig. 1, IPSSGB identifies 70% of important features when the target FDR is 0.1, while IPSSRF identifies 40% and the remaining methods identify close to none. IPSSGB’s TPR even surpasses the TPR of methods without error control in almost all experiments despite having far fewer false positives. With an average runtime of <15 s in all experiments, IPSSGB is also one of the fastest methods.

Among the other methods with theoretical error control, IPSSRF performs well in terms of identifying true positives while controlling false positives, though not as well as IPSSGB. IPSSL1, whose parametric assumptions are violated, performs poorly, failing to control false positives at target FDR levels. It also identifies essentially no true positives in the Gaussian experiments. SSBoost is overly conservative, undershooting the target FDR at the expense of identifying few true positives. This is partly due to the weakness of the efp scores used by SSBoost relative to those used by IPSS (Melikechi and Miller 2024).

All versions of model-X knockoffs underperform IPSSGB and IPSSRF in all experiments. This is even true in the Gaussian setting, where the known joint distribution of the features was used to implement these methods (recall that model-X knockoffs requires knowledge of the joint distribution, while IPSS does not). With the exception of KOL and KORF in the P=5000 and, to a lesser extent, P=2000 RNA-seq experiments, all of these methods control the FDR at target levels. KORF is the only knockoffs-based method that identifies essentially any true positives in the Gaussian experiments. DeepPINK, which combines knockoffs with deep neural networks, has the lowest TPR out of every method in almost all experiments. This agrees with our earlier observation that deep learning typically underperforms tree-based methods on tabular data and requires tens of thousands of samples for competitive performance (Grinsztajn *et al.* 2022).

The absence of error control for Boruta and Vita is clearly apparent in Fig. 2 and Fig. 2, available as supplementary data at Bioinformatics online. Both methods usually have FDRs over 0.75, far surpassing other methods. Despite this, Boruta and Vita almost always identify fewer true positives than IPSSGB when the target FDR is greater than 0.2 or, in some cases, even 0.1. Boruta and Vita are also slower than other methods, and this disparity grows with the number of features (Tables 3 and 4, available as supplementary data at Bioinformatics online).

VSURF usually has a lower FDR than Boruta and Vita, but still underperforms IPSSGB. Its excessive runtimes prevented us from including it in the P=2000 and 5000 RNA-seq experiments. Several other methods are also omitted, namely KOBT, RFEGB, and RFHT. Briefly, KOBT far exceeded target FDRs despite extensive tuning, while RFEGB and RFHT had FDRs over 0.8 and extremely long runtimes. For details, see Sections 2 and 3, available as supplementary data at Bioinformatics online.

## 4 Cancer studies

We study ovarian cancer and glioma (a type of brain cancer). Both studies include multiple substudies in which the features are either genes, measured by RNA-seq, or microRNAs (miRNAs). The response variables are either *prognosis* (whether the patient was alive at last follow-up), *tumor purity* (the proportion of cancerous cells in a tissue sample), or the expression level of a particular gene or miRNA. All data are from The Cancer Genome Atlas (Weinstein *et al.* 2013) and were downloaded from LinkedOmics (Vasaikar *et al.* 2018). Additional details and results are in Section 4, available as supplementary data at Bioinformatics online.

We assess feature selection performance by (i) performing literature searches and (ii) assessing predictive performance using cross-validation, detailed in Sections 4.3 and 4.4, available as supplementary data at Bioinformatics online, respectively. The literature search results show that IPSSGB and IPSSRF consistently identify more known important features at lower target FDRs than other methods. In Table 6, available as supplementary data at Bioinformatics online, for example, IPSSGB and IPSSRF identify 8 and 6 miRNAs, respectively, all but one of which have been implicated in ovarian cancer prognosis. In contrast, IPSSL1 identifies 4 miRNAs, missing the three most significant ones, while KOGLM, KORF, KOL1, DeepPINK, and SSBoost select no miRNAs at all, even when the target FDR is 0.5.

In the RNA-seq and glioma prognosis study (Table 10, available as supplementary data at Bioinformatics online), only IPSSGB identifies the key oncogene FOXM1 (Raychaudhuri and Park 2011), and only IPSSGB, IPSSRF, and KORF identify WEE1, which is also known to play a significant role in glioma outcomes (Music *et al.* 2016). Furthermore, IPSSGB and IPSSRF are more confident in their selections, assigning WEE1 *q*-values of 0.10 and 0.06, respectively, while KORF does not select WEE1 until the target FDR is reduced to 0.44. More generally, Table 10, available as supplementary data at Bioinformatics online shows that IPSSGB, IPSSRF, and, to a lesser extent, IPSSL1, tend to identify genes supported by the glioma literature while avoiding genes with little or no known connection. In contrast, KORF and especially KOL1 select many genes with limited or no literature support, while KOGLM, DeepPINK, and SSBoost select no genes at all.

Briefly, our cross-validation (CV) studies proceed as follows. In each of 20 CV steps, one group of patients is set aside (the test set), and a set of features is selected by each method using the data in the remaining groups (the training set). Next, for each method we construct three predictive models—a linear model, a random forest model, and a gradient boosting model—using only the features selected by that method on the training data. Each model is then used to predict responses from the test set, and the smallest of the three prediction errors is recorded (we use mean squared error for regression and 1−accuracy for classification). All three models are implemented so that no method has an inherent advantage over another. For example, features selected by IPSSL1 may be better suited to linear model predictions than those selected by IPSSGB, while those selected by IPSSGB may be better suited to gradient boosting predictions than the ones selected by IPSSL1.

**Figure 3.**
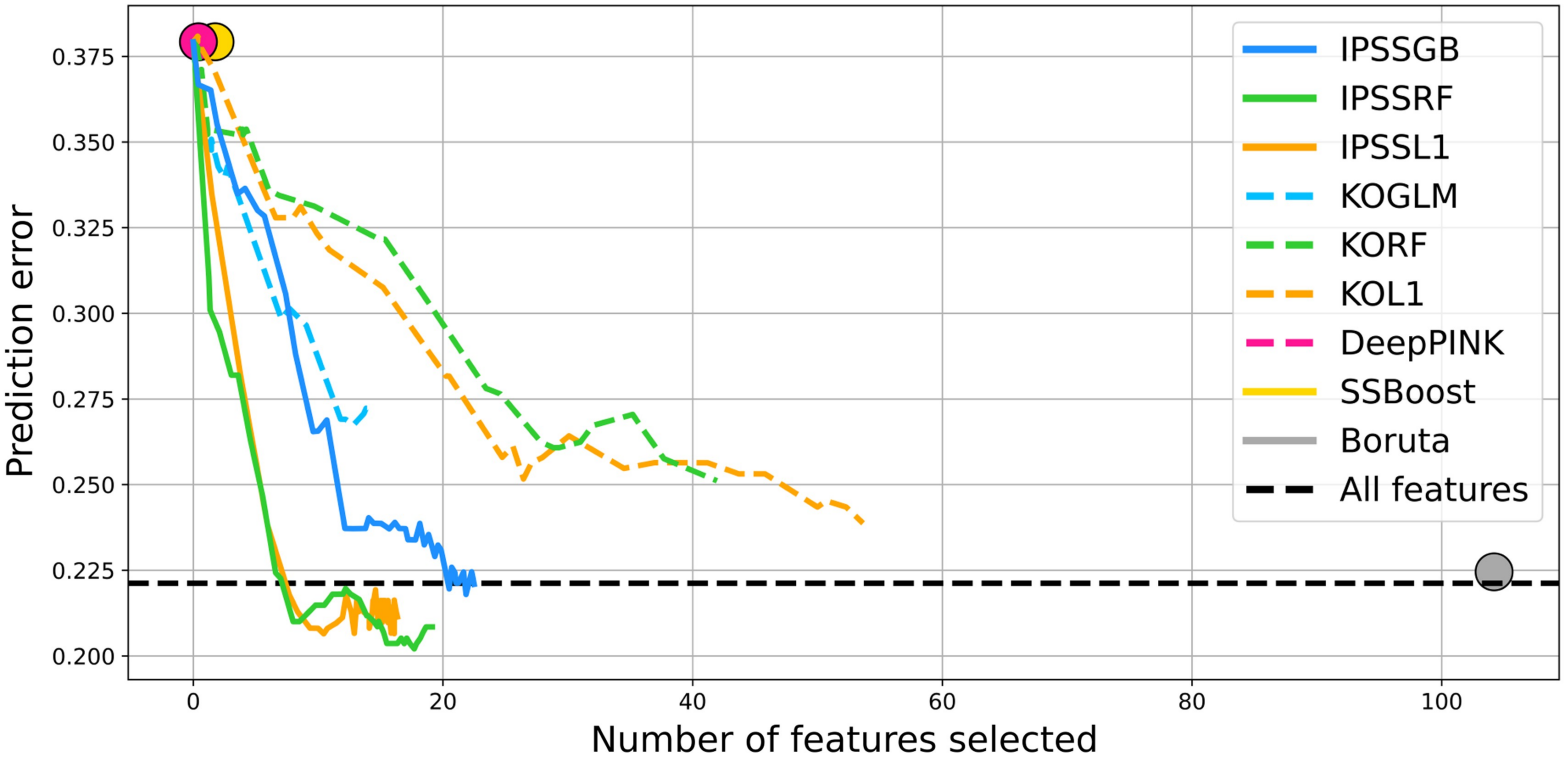
*RNA-seq and glioma prognosis*. The horizontal and vertical axes show the average number of genes selected and the average prediction error over the 20 cross-validation steps, respectively. Curves for each method are obtained by varying the target FDR between 0 and 0.5. DeepPINK and SSBoost selected no genes and are therefore shown by single points rather than curves. Boruta is also indicated by a single point because it does not have FDR control parameters. The dashed black line shows the average error when using all *P* = 10 058 genes to predict prognosis.

Figure 3 shows the results from our RNA-seq and glioma prognosis CV study. On average, the top 20 genes selected by IPSSGB yield the same prediction error as the full model that uses all 10, 058 genes. IPSSRF and IPSSL1 achieve the smallest prediction errors overall, and only use 10–20 genes to do so. KORF and KOL1 have higher predictive errors despite using more than 40 selected genes, and Boruta, which selects over 100 genes, has an average error similar to that of the full model. Vita (not shown) selects over 500 genes on average and has an average prediction error of 0.22, and DeepPINK and SSBoost select no genes at all, even when the target FDR is 0.5.

## 5 Discussion

We have demonstrated that IPSSRF and IPSSGB achieve superior results in terms of false positive control, true positive detection, and computation time. More broadly, IPSS for thresholding is a general framework whose theory and implementation apply to arbitrary importance scores. For instance, examples of other scores include *P*-values (with smaller *P*-values indicating greater importance), Shapley values (used to quantify the contribution of individual features to neural networks and other machine learning models), and loadings in principal components analysis (which quantify the contribution of each feature to a given principal component). The main practical limitation to consider is the cost of computing the relevant importance scores, since IPSS must compute these scores on multiple subsamples of the data.

We have also introduced efp scores and shown that, in addition to controlling E(FP), they can control the FDR and estimate *q*-values. Storey (2003) showed that *q*-values admit a Bayesian interpretation, suggesting a link between IPSS and Bayesian feature selection that could be an interesting line of future work.

Finally, a more ambitious goal is to extend IPSS to unsupervised feature selection problems (that is, feature selection when there is no response variable) and non-iid data. The PCA-based scores mentioned above provide at least one way to apply IPSS in an unsupervised setting. Developing a rigorous approach to IPSS for non-iid data could provide novel methods for nonparametric feature selection with false discovery control for networks and spatially or temporally-indexed data.

## Supplementary Material

btaf299_Supplementary_Data
