# Supplementary material for: Nonparametric IPSS: fast, flexible feature selection with false discovery control
Source: Bioinformatics. 2025 May 13;41(5):btaf299. doi: 10.1093/bioinformatics/btaf299 (PMC12119134; doi:10.1093/bioinformatics/btaf299)
Supplement: btaf299_Supplementary_Data [file btaf299_supplementary_data.pdf]

Supplementary material for "nonparametric IPSS: fast, flexible feature selection with false discovery control"

Omar Melikechi[1], David B. Dunson[2], and Jeffrey W. Miller[1]

We provide further details about IPSS (Section S1), describe the feature selection methods considered in this work (Section S2), and present results from our simulation studies (Section S3), cancer studies (Section S4), and sensitivity analyses of the IPSS parameters (Section S5).

S1. IPSS details

We provide an algorithm that implements integrated path stability selection (IPSS) for feature importance scores (Section S1.1), elaborate on the theory of IPSS and its connection to efp scores (Section S1.2), and describe the IPSS parameters in greater detail (Section S1.3).

S1.1. Algorithm. Algorithm S1, discussed in Section 2.2, implements IPSS for feature importance scores. The number of grid points used to evaluate the integrals in Algorithm S1 is always $K = 100$. Like many of the other IPSS parameters, K is inconsequential provided it is sufficiently large; in our experience, values greater than 25 suffice. This is because the function $f(x) = (2x-1)^3\,\mathbb{1}(x \geq 0.5)$, the paths $\lambda \mapsto \hat{\pi}_j(\lambda)$ (which are monotonically increasing functions of λ), the quantity $\mathcal{I}(\Lambda)$, and the family of measures $\mu_\delta(d\lambda) = z_\delta^{-1}\lambda^{-\delta}d\lambda$ are all very numerically stable.

S1.2. IPSS theory and efp scores. Given the estimated selection probabilities $\hat{\pi}_j$, an interval $\Lambda \subseteq (0,\infty)$, a probability measure μ on Λ, a function $f : [0,1] \to \mathbb{R}$, and a threshold τ, Melikechi and Miller (2024) define the set of features selected by IPSS by

$$\hat{S}_{\mathrm{IPSS}}(\tau) = \Big\{ j : \textstyle\int_\Lambda f(\hat{\pi}_j(\lambda))\mu(d\lambda) \geq \tau \Big\}. \tag{S1.1}$$

The integral incorporates information about the selection probabilities over all of Λ, eliminating the need to select features based on individual λ values. The function f transforms the selection probabilities for better performance. Only certain choices of f are known to yield valid efp scores. Melikechi and Miller (2024) prove the following result (see Theorem 4.1 therein). Let $q(\lambda) = \mathrm{E}|\hat{S}_\lambda(Z_{1:\lfloor n/2\rfloor})|$ be the expected number of features selected by $\hat{S}_\lambda$ on half the data and, for $m \in \mathbb{N}$, define $h_m(x) = (2x-1)^m\,\mathbb{1}(x \geq 0.5)$. For any Λ and μ, if

$$\max_{j \in S^c} \mathbb{P}\Big(j \in \bigcap_{b=1}^{m'} \big(\hat{S}_\lambda(Z_{A_{2b-1}}) \cap \hat{S}_\lambda(Z_{A_{2b}})\big)\Big) \leq (q(\lambda)/p)^{2m'}, \tag{S1.2}$$

for all $\lambda \in \Lambda$ and $m' \in \{1,\ldots,m\}$, then IPSS with $f = h_m$ satisfies

$$\mathrm{E}|\hat{S}_{\mathrm{IPSS}}(\tau) \cap S^c| \leq \frac{\mathcal{I}_m(\Lambda)}{\tau} \tag{S1.3}$$

[1]Department of Biostatistics, Harvard T.H. Chan School of Public Health, Boston, MA
[2]Department of Statistical Science, Duke University, Durham, NC

Algorithm S1 Integrated path stability selection for feature importance scores

Input: Data $Z_{1:n}$, importance function Φ, number of grid points K and iterations B, probability measure μ, function f (default $f(x) = (2x-1)^3\,\mathbb{1}(x \geq 0.5)$), and cutoff C (default $C = 0.05$).

(Optional) Preselect features, as described in Section S2.1.
for $b = 1, \ldots, B$ **do**
    Randomly select $A_{2b-1}, A_{2b} \subseteq \{1, \ldots, n\}$ with $A_{2b-1} \cap A_{2b} = \varnothing$ and $|A_{2b-1}| = |A_{2b}| = \lfloor n/2 \rfloor$.
    Evaluate $\Phi_{Z_{A_{2b-1}}}(j)$ and $\Phi_{Z_{A_{2b}}}(j)$ for $j = 1, \ldots, p$.
end for
Set $\lambda_{\max} = \max\{\Phi_{Z_{A_b}}(j) : 1 \leq b \leq 2B,\ 1 \leq j \leq p\}$.
Define a λ grid with upper bound $\lambda_{\max}$, e.g., $\lambda_{\max} = \lambda_0 > \lambda_1 > \cdots > \lambda_K = \lambda_{\max}/10^8$.
Initialize $\lambda_{\min} \leftarrow \lambda_{\max}$ and $k \leftarrow 0$.
while $\mathcal{I}([\lambda_{\min}, \lambda_{\max}]) < C$ **do**
    $\hat{S}_\lambda(Z_{A_b}) = \{j : \Phi_{Z_{A_b}}(j) \geq \lambda\}$ for $b = 1, \ldots, 2B$.
    $\lambda_{\min} \leftarrow \lambda_{k+1}$ followed by $k \leftarrow k + 1$.
end while
$\Lambda \leftarrow [\lambda_{\min}, \lambda_{\max}]$.
Evaluate estimated selection probability $\hat{\pi}_j(\lambda) = \frac{1}{2B}\sum_{b=1}^{2B} \mathbb{1}\big(j \in \hat{S}_\lambda(Z_{A_b})\big)$ for $j = 1, \ldots, p$.
Evaluate the integral $\int_\Lambda f(\hat{\pi}_j(\lambda))\mu(d\lambda)$ for $j = 1, \ldots, p$.

Output: $\texttt{efp}_{Z_{1:n}}(j) = \mathcal{I}(\Lambda) / \int_\Lambda f(\hat{\pi}_j(\lambda))\mu(d\lambda)$ for $j = 1, \ldots, p$.

for a constant $\mathcal{I}_m(\Lambda)$ whose explicit form is given in Theorem 4.1 in Melikechi and Miller (2024). Thus, setting $\texttt{efp}_{Z_{1:n}}(j) = \mathcal{I}_m(\Lambda) / \int_\Lambda h_m(\hat{\pi}_j(\lambda))\mu(d\lambda)$ and $\hat{S}(t) = \{j : \texttt{efp}_{Z_{1:n}}(j) \leq t\}$, we have

$$\hat{S}(t) = \left\{ j : \frac{\mathcal{I}_m(\Lambda)}{\int_\Lambda h_m(\hat{\pi}_j(\lambda))\mu(d\lambda)} \leq t \right\} = \left\{ j : \int_\Lambda h_m(\hat{\pi}_j(\lambda))\mu(d\lambda) \geq \frac{\mathcal{I}_m(\Lambda)}{t} \right\} = \hat{S}_{\text{IPSS}}\left(\frac{\mathcal{I}_m(\Lambda)}{t}\right)$$

and hence

$$\mathrm{E}(\mathrm{FP}(t)) = \mathrm{E}|\hat{S}(t) \cap S^c| = \mathrm{E}\big|\hat{S}_{\text{IPSS}}\big(\tfrac{\mathcal{I}_m(\Lambda)}{t}\big) \cap S^c\big| \leq t,$$

where the inequality holds by Equation S1.3.

The above derivation shows how valid efp scores for IPSS with $f = h_m$ are obtained under the conditions of Theorem 4.1 in Melikechi and Miller (2024). In particular, we see that by defining efp scores as above, the set $\hat{S}(t) = \{j : \texttt{efp}_{Z_{1:n}}(j) \leq t\}$ is identical to $\hat{S}_{\text{IPSS}}(\tau)$ when $\tau = \mathcal{I}_m(\Lambda)/t$. The main condition of the theorem, Equation S1.2, upper bounds the maximum probability that an unimportant feature is simultaneously selected on both halves of the data, $Z_{A_{2b-1}}$ and $Z_{A_{2b}}$, in m' independent tries; see Melikechi and Miller (2024) for details. The following result, part of Theorem 4.2 in Melikechi and Miller (2024), gives the form of $\mathcal{I}_3(\Lambda)$, which corresponds to the function $f = h_3$ that is used for IPSS throughout the main text.

Theorem S1.1. *Let μ be a probability measure on $\Lambda \subseteq (0, \infty)$, let $\tau \in (0, 1]$, and define $\hat{S}_{\text{IPSS}}(\tau)$ as in Equation* S1.1 *with $f = h_3$. If Equation* S1.2 *holds for all $\lambda \in \Lambda$ and $m' \in \{1, 2, 3\}$, then*

$$\mathrm{E}(\mathrm{FP}(\tau)) \leq \frac{1}{\tau}\int_\Lambda \left(\frac{q(\lambda)^2}{B^2 p} + \frac{3q(\lambda)^4}{Bp^3} + \frac{q(\lambda)^6}{p^5} \right)\mu(d\lambda), \qquad \text{(S1.4)}$$

where $\mathrm{E}(\mathrm{FP}(\tau)) = \mathrm{E}|\hat{S}_{\text{IPSS}}(\tau) \cap S^c|$ is the expected number of false positives selected by IPSS.

For some intuition about the bound in Equation S1.4, observe that taking $B \to \infty$ yields $\mathrm{E}(\mathrm{FP}(\tau)) \leq \tau^{-1}\int_\Lambda \big(q(\lambda)^6/p^5\big)\mu(d\lambda)$. In comparison, other versions of stability selection upper bound $\mathrm{E}(\mathrm{FP}(\tau))$

by $q(\lambda)^2/p$ (Meinshausen and Bühlmann, 2010; Shah and Samworth, 2013), which is orders of magnitude larger than $q(\lambda)^6/p^5$ when $q(\lambda) \ll p$, as is often the case for most values of λ in Λ. This and the contribution of B in the denominators in Equation S1.4 largely explain the strength of the IPSS bound in Theorem S1.1 relative to previous bounds, and hence the tightness of the efp scores of IPSS with $f = h_3$ relative to the efp scores of other versions of stability selection. Further theoretical and empirical comparisons between Equation S1.4 and other stability selection bounds are available in Melikechi and Miller (2024).

S1.3. Parameters. Table S1 shows the default IPSS parameters used for `IPSSGB` and `IPSSRF` throughout this work (default gradient boosting and random forest parameters are in Section S2.2.1 and Section S2.2.2). As noted above, our choice of function $f(x) = (2x-1)^3\,\mathbb{1}(x \geq 0.5)$ is determined by the availability and strength of the theoretical bound in Theorem S1.1. Similarly, the choice of $\lfloor n/2 \rfloor$ samples used to construct the selection probabilities $\hat{\pi}_j(\lambda)$ is required for stability selection theorems (not just Theorem S1.1) to hold, and it is unclear how to adapt their proofs to accommodate other sample sizes. Thus, f and $\lfloor n/2 \rfloor$ are theoretically determined rather than free parameters.

The interval $\Lambda = [\lambda_{\min}, \lambda_{\max}]$ is determined by setting $\lambda_{\max}$ large enough that no features are selected (see, for example, Line 6 in Algorithm S1), and setting $\lambda_{\min}$ such that the integral

$$\mathcal{I}(\Lambda) = \int_\Lambda \left(\frac{q(\lambda)^2}{B^2 p} + \frac{3q(\lambda)^4}{Bp^3} + \frac{q(\lambda)^6}{p^5}\right)\mu(d\lambda),$$

in Equation S1.4 is equal to a fixed cutoff C. As noted in the main text, we always use $C = 0.05$, but results are largely independent of this choice (Figures S12 and S13). Figures S14 and S15 show IPSS is also robust to the parameter δ that determines the measure $\mu_\delta(d\lambda) = z_\delta^{-1}\lambda^{-\delta}d\lambda$, where the normalizing constant z_δ is easily computed in closed form (Melikechi and Miller, 2024). The probability measure μ_1 averages over Λ on a log scale, while μ_0 averages on a linear scale.

The insignificance of C and δ is unsurprising: Intuitively, the efp scores for IPSS depend primarily on f and the integrand in $\mathcal{I}(\Lambda)$. The actual value of $\mathcal{I}(\Lambda)$ is much less important since the efp scores depend on the relative quantities $\mathcal{I}(\Lambda)/\int_\Lambda f(\hat{\pi}_j(\lambda))\mu(d\lambda)$ rather than on $\mathcal{I}(\Lambda)$ itself. Since C and μ only affect the value of the bound, not the integrand, they contribute little to the actual performance of IPSS, as indicated by the sensitivity analyses in Section S5.

**Method**	B	C	$f(x)$	δ_{reg}	δ_{class}
`IPSSGB`	100	0.05	$(2x-1)^3\,\mathbb{1}(x \geq 0.5)$	1.25	1
`IPSSRF`	50	0.05	$(2x-1)^3\,\mathbb{1}(x \geq 0.5)$	1.25	1.25

Table S1. *Default IPSS parameters.* Both `IPSSGB` and `IPSSRF` always use $C = 0.05$ and $f(x) = (2x-1)^3\,\mathbb{1}(x \geq 0.5)$. `IPSSRF` uses $B = 50$ to reduce runtimes without any noticeable difference in selection performance. The parameters δ_{reg} and δ_{class} determine the measure μ_δ in regression and classification problems, respectively.

S2. Overview of methods and implementation details

We describe the preselection procedure used to improve feature selection (Section S2.1) and provide implementation details for each feature selection method considered in this work (Section S2.2).

S2.1. Preselection. Many feature selection methods employ some form of screening, or *preselection*, as an initial step in the selection process. This can be especially helpful in high dimensions for increasing power and reducing runtimes. For many of the methods in Section S2.2, we preselect features by running a randomized importance function on the full dataset three times—that is, computing $\Phi_{Z_{1:n}}$ three times—and keeping only the p_{pre} features with the largest average scores across all three trials. For example, preselection for `IPSSRF` entails fitting three random forests to the full dataset and keeping the p_{pre} features with the largest average importance scores.

For IPSS, this preselection step does not affect the theoretical control on E(FP) since $|\hat{S}_{\text{IPSS,pre}} \cap S^c| = |\hat{S}_{\text{IPSS,pre}} \cap S^c_{\text{pre}}|$, where $\hat{S}_{\text{IPSS,pre}}$ are the features selected by IPSS using only the preselected features, and S^c_{pre} are the preselected features in S^c. Of course, preselection risks discarding important features, potentially increasing the number of false negatives. In practice, however, we find that preselection actually helps IPSS, stability selection, and model-X knockoffs identify more true positives while still controlling false discoveries. This is because preselection gets rid of many noisy features, making it easier for these methods to detect the true signal.

In Section S2.2, we describe the preselection parameters for each method, which were determined by extensive testing on simulated data. We do not apply preselection when implementing `Boruta`, `RFEGB`, `RFHT`, `Vita`, and `VSURF` since these methods include their own internal screening steps.

S2.2. Methods. We describe how each method in Table S2 is implemented in this work.

Method	Package	Error control	Base method	Non-default settings
`IPSSGB`	*ipss* (Python)	✓	Boosting	—
`IPSSRF`	*ipss* (Python)	✓	Random forest	—
`IPSSL1`	*ipss* (Python)	✓	Lasso	—
`KOGLM`	*knockoff* (R)	✓	GLM	—
`KOL1`	*knockoff* (R)	✓	Lasso	—
`KORF`	*knockoff* (R)	✓	Random forest	—
`DeepPINK`	*knockpy* (Python)	✓	Neural network	—
`SSBoost`	*XGBoost* (Python) with *stabs* (R)	✓	Boosting	`assumption` = r−concave $\tau = 0.75$
`KOBT`	*KOBT* (R)	✓	Boosting	`num` = 100, `bound` = 200, `type` = shrink
`RFHT`	*rfvimptest* (R)	✓	Random forest	—
`Boruta`	*Boruta* (R)	✗	Random forest	—
`RFEGB`	*scikit-learn* (Python)	✗	Boosting	—
`Vita`	*vita* (R)	✗	Random forest	p-value threshold = 0
`VSURF`	*VSURF* (R)	✗	Random forest	`VSURF_pred`

TABLE S2. *Feature selection methods.* Software packages are listed with the language used to implement them in parentheses. Details about each method, including descriptions of their non-default settings, are in Sections S2.2.1–S2.2.7. For methods with no non-default settings, we use the default settings in their respective packages.

S2.2.1. `IPSSGB`. The IPSS-related parameters used to implement `IPSSGB` are in Table S1. For preselection, we use gradient boosting as the baseline selection algorithm and set $p_{\text{pre}} = 100$. We implement gradient boosting using `XGBoost` (Chen and Guestrin, 2016). All `XGBoost` parameters

are set to their default values except for two changes: The proportion of features considered when splitting each node (called `colsample_bynode` in `XGBoost` and often `mtry` elsewhere) is changed from 1 to 1/3, and the maximum depth of each tree (`max_depth`) is changed from 6 to 1, making each tree a stump. The latter change significantly improved the performance of `IPSSGB`, both in terms of speed and feature selection.

S2.2.2. `IPSSRF`. The IPSS-related parameters used to implement `IPSSRF` are in Table S1. For preselection, we use random forests as the baseline selection algorithm and set $p_{\text{pre}} = 100$. We implement random forests using `scikit-learn` (Pedregosa et al., 2011). All random forest parameters are set to their default values except for two changes: The proportion of features considered when splitting each node (called `max_features` in `scikit-learn`) is changed from 1 to 1/10, and the number of trees used to build each random forest (`n_estimators`) is changed from 100 to 50. These changes improved the efficiency of `IPSSRF` without sacrificing its feature selection performance.

S2.2.3. `IPSSL1`. As discussed in the main text, `IPSSL1` is a parametric version of IPSS based on ℓ^1-regularization (Melikechi and Miller, 2024). For regression, the baseline algorithm is lasso (Tibshirani, 1996), and for classification, it is ℓ^1-regularized logistic regression (Friedman et al., 2010). All parameters are set to their default values in the `ipss` Python package: https://pypi.org/project/ipss/.

S2.2.4. Model-X knockoffs (`KOGLM`, `KOL1`, `KORF`, `DeepPINK`, `KOBT`). Model-X knockoffs work by first constructing *knockoffs* $\tilde{X} = (\tilde{X}_1, \dots, \tilde{X}_p)$ of the original features $X = (X_1, \dots, X_p)$. By definition, $\tilde{X}$ must satisfy (i) the joint distribution of $(X, \tilde{X})$ is invariant under pairwise exchanges of the original features X_j and their corresponding knockoffs $\tilde{X}_j$, and (ii) $\tilde{X}$ is conditionally independent of the Y given X (see Definition 2 in Candes et al. (2018)). Once knockoffs are constructed, feature importance scores—called *feature statistics* in the knockoffs literature—are computed for all of the original and knockoff features and subsequently used to select original features in a way that controls the FDR. Like IPSS, any feature importance function can be used.

As noted in the main text, model-X knockoffs requires knowledge of the joint distribution of X. In our multivariate Gaussian simulations in Section 3, all of the model-X knockoffs methods are implemented using the true, known joint distribution. In the remaining examples, where the joint distribution of X is not known, we use the default methods for constructing approximate model-X knockoffs (for example, second-order Gaussian knockoffs in the `knockoff` R package).

`KOGLM`, `KOL1`, and `KORF` are all implemented using the R package `knockoff`. `KOGLM` uses feature importance scores from a generalized linear model (GLM); we find it performs best when using random forests for preselection with $p_{\text{pre}} = 200$. `KOL1` uses feature importance scores from lasso (regression) or ℓ^1-regularized logistic regression (classification). Like `IPSSL1`, we use lasso (or logistic regression) for preselection, with $p_{\text{pre}} = 200$. `KORF` uses feature importance scores from random forests; like `KOGLM`, we find it performs best when using random forests for preselection with $p_{\text{pre}} = 200$.

`DeepPINK` uses deep neural networks to construct feature importance scores. We implement it using the Python package `knockopy` and random forests for preselection with $p_{\text{pre}} = 100$.

`KOBT` is implemented with the R package `KOBT`. It uses boosted tree models to construct importance scores. We tested `KOBT` numerous times on simulated data with many different tuning and preselection parameters (including no preselection), but found that `KOBT` consistently and dramatically exceeded its target FDR. For this reason, we omit its results from all plots and tables in this work.

Finally, we tested all of the model-X knockoffs methods without using preselection. In these cases, power was often extremely low and the runtimes were much longer.

S2.2.5. `SSBoost`. The closest method to `IPSSGB` in terms of its underlying approach is that of Hofner et al. (2015), referred to here as `SSBoost`. Unlike `IPSSGB`—which uses importance scores from gradient boosting—`SSBoost` applies stability selection to choose the number of features used per boosting run. Furthermore, `IPSSGB` uses IPSS to construct efp scores, whereas `SSBoost` uses a version of stability selection introduced by Shah and Samworth (2013). This is perhaps the most significant difference since the efp scores for IPSS have much tighter bounds than those for other forms of stability selection (Melikechi and Miller, 2024). From a practical standpoint, this causes other versions of stability selection to identify fewer important features than IPSS.

Hofner et al. (2015) provide code for `SSBoost` that combines the R packages `mboost` (Hofner et al., 2014) and `stabs` (Hofner and Hothorn, 2017) in the form of a worked example, but the `mboost` implementation of boosting was prohibitively slow in the dimensions we consider. By adapting their code to use `XGBoost` in place of `mboost` for boosting and by preselecting features using gradient boosting with $p_{\text{pre}} = 150$, we were able to reduce `SSBoost` runtimes considerably with no apparent change in results. The `XGBoost` parameters used for `SSBoost` are the same as those used for `IPSSGB` (Section S2.2.1). For the stability selection part of `SSBoost`, we use the default parameters in `stabs`, and the selection threshold is set to $\tau = 0.75$, which is the middle of the interval $(0.6, 0.9)$ recommended by Meinshausen and Bühlmann (2010).

S2.2.6. `RFHT`. We tested `RFHT` (Coleman et al., 2022), which achieves theoretical error control by using random forests for hypothesis testing. However, one test run with default parameters on simulated data with 500 samples, 500 features, and 20 true features took over 51 minutes, returning 15 true positives and 43 false positives. By contrast, `IPSSGB` with a target FDR of 0.2 took 11 seconds and returned 10 true positives and 0 false positives on the same data. This method is omitted from our studies because its performance does not appear to justify its excessive runtime.

S2.2.7. Methods without false discovery control (`Boruta`, `RFEGB`, `Vita`, `VSURF`). `Boruta`, `Vita`, and `VSURF` are implemented using R packages of the same names. `Boruta` is run with default parameters. For `Vita`, we set the p-value threshold to 0. For `VSURF`, we use the function `VSURF_pred` rather than `VSURF_interp` to select the final set of features (Genuer et al., 2010). Both of these choices favor sparsity, which aligns well with our simulation designs. As noted in the main text, `VSURF` is too computationally expensive to include in our $p = 2000$ and 5000 simulation studies; see also Table S3.

We implement `RFEGB` by combining `XGBoost` and `scikit-learn`. On simulated Gaussian data with $n = 250$ and $p = 500$, one run of `RFEGB` took over 12 minutes when removing five features per iteration (the default is one feature removed per iteration, which takes approximately 5 times as long). For comparison, `IPSSGB` ran in 5 seconds on the same data and had far fewer false positives and similar power. Due to its high computational cost, poor performance, and many tuning parameters (which is true of recursive feature elimination in general), `RFEGB` is largely omitted from this work.

S3. Simulation results and details

We present additional simulation results (Section S3.1) and RNA-seq simulation details (Section S3.2).

S3.1. Additional simulation results. Figure S1 shows the $n = 250$ multivariate Gaussian simulation results, described in Section 3.2. Figure S2 shows the $p = 500$, 2000, and 5000 RNA-seq simulation results for classification, described in Section 3.3. Tables S3 and S4 show the average runtimes of each method in each simulation experiment.

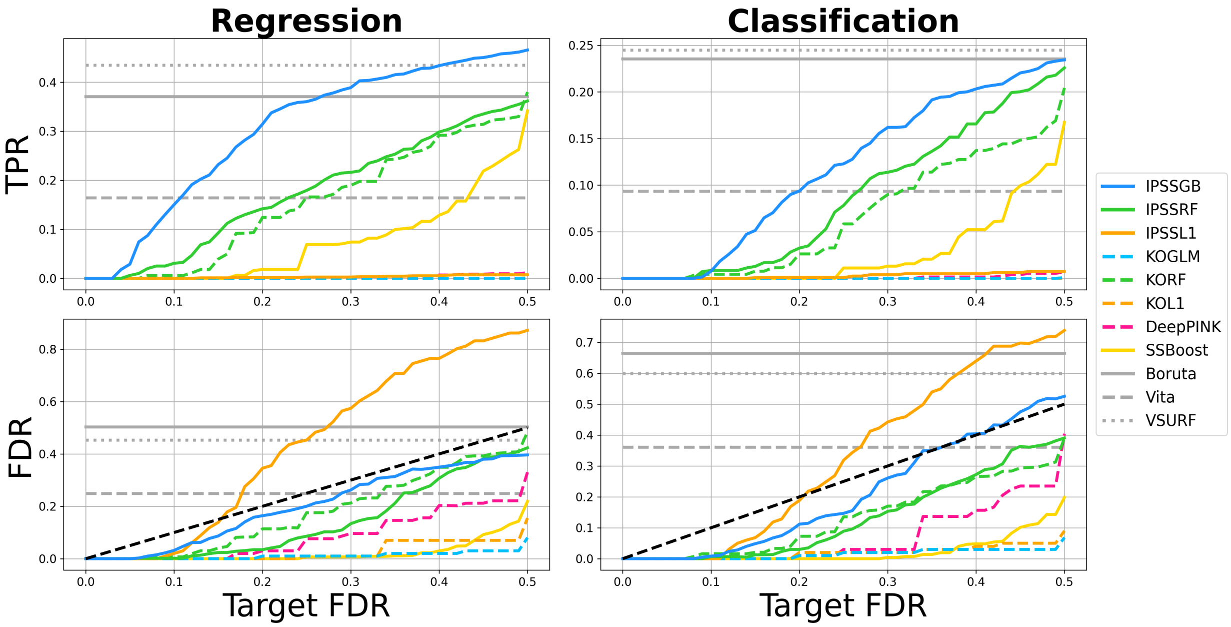

Figure S1. *Gaussian simulation results* $(n = 250)$. See Figure 1 for details.

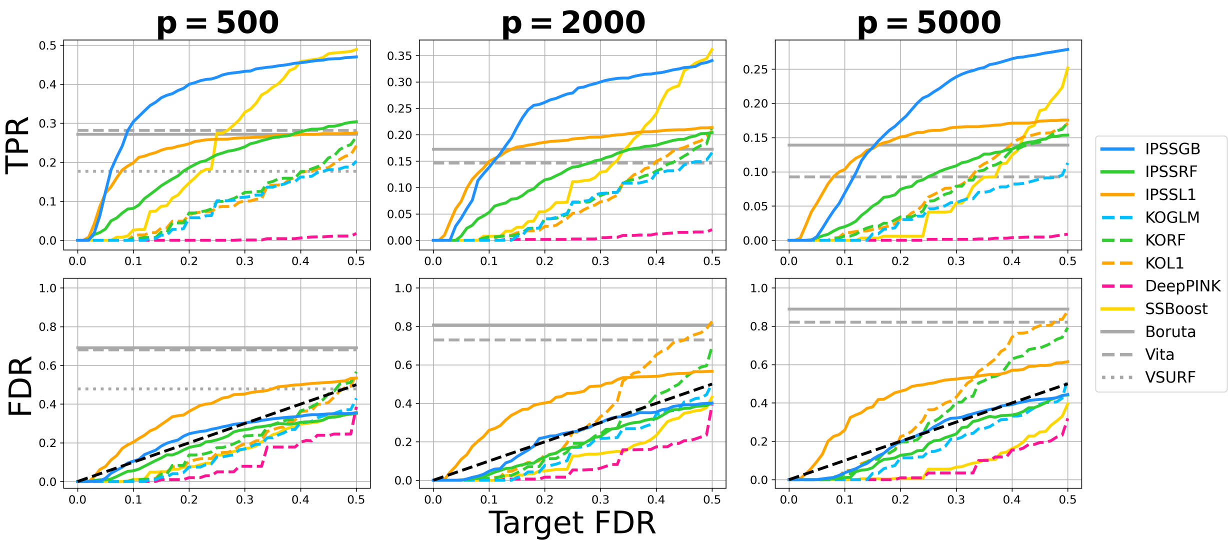

Figure S2. *RNA-seq simulation results (classification)*. See Figure 2 for details.

**Method**	MG($n = 250$)	MG($n = 500$)	RNA($p = 500$)	RNA($p = 2000$)	RNA($p = 5000$)
`IPSSGB`	4.4 (0.11)	6.3 (0.01)	6.7 (0.23)	9.0 (0.43)	14.6 (0.45)
`IPSSRF`	4.5 (0.05)	7.5 (0.10)	7.2 (0.14)	10.8 (0.29)	19.6 (0.63)
`IPSSL1`	1.6 (0.23)	1.0 (0.05)	4.0 (0.91)	3.1 (0.66)	6.4 (0.76)
`KOGLM`	10.8 (0.92)	12.4 (0.91)	10.0 (1.92)	12.6 (1.50)	19.2 (1.66)
`KOL1`	11.2 (0.88)	12.0 (1.04)	7.5 (1.27)	6.7 (0.93)	7.2 (1.16)
`KORF`	12.8 (1.16)	14.3 (1.01)	4.2 (0.50)	7.4 (0.22)	13.9 (0.50)
`DeepPINK`	5.0 (3.55)	6.0 (2.79)	5.9 (4.17)	8.8 (0.21)	15.4 (0.42)
`SSBoost`	4.1 (0.04)	6.7 (0.03)	6.7 (0.04)	8.1 (0.15)	13.7 (0.08)
`Boruta`	42.2 (3.09)	120.7 (7.93)	42.0 (5.96)	57.0 (6.51)	84.5 (6.66)
`Vita`	9.5 (0.19)	24.2 (0.40)	20.6 (0.47)	83.5 (4.09)	195.3 (4.34)
`VSURF`	196.8 (8.16)	548.6 (23.68)	286.7 (72.36)	—	—
`RFHT`	—	3087*	—	—	—
`RFEGB`	749*	—	—	—	—

Table S3. Average runtimes (in seconds) over 100 trials for each regression experiment. MG stands for Multivariate Gaussian. Standard deviations are in parentheses. Recall that $p = 500$ in the MG experiments and $n = 500$ in the RNA experiments, and that `VSURF` was too computationally expensive to include when $p = 2000$ and 5000. *As discussed in Sections S2.2.6 and S2.2.7, `RFHT` and `RFEGB` are largely omitted due to their poor selection performance and excessive runtimes in initial tests (shown in the table). Hence, their remaining entries are blank.

**Method**	MG($n = 250$)	MG($n = 500$)	RNA($p = 500$)	RNA($p = 2000$)	RNA($p = 5000$)
`IPSSGB`	4.3 (0.04)	6.5 (0.11)	6.6 (0.07)	7.5 (0.20)	14.3 (0.42)
`IPSSRF`	4.2 (0.02)	7.0 (0.05)	6.8 (0.10)	9.9 (0.22)	17.7 (0.49)
`IPSSL1`	5.9 (0.15)	6.0 (0.22)	8.4 (0.86)	9.3 (1.17)	13.1 (1.25)
`KOGLM`	10.6 (0.10)	12.0 (0.14)	9.3 (1.57)	11.8 (1.67)	17.4 (1.64)
`KOL1`	10.7 (0.12)	11.9 (0.21)	7.2 (1.13)	6.0 (0.83)	6.3 (0.80)
`KORF`	10.1 (0.95)	12.1 (0.17)	3.1 (0.10)	5.9 (0.18)	11.3 (0.38)
`DeepPINK`	4.7 (0.29)	5.7 (0.28)	5.2 (0.10)	8.0 (0.24)	13.3 (0.43)
`SSBoost`	4.0 (0.01)	6.6 (0.04)	6.7 (0.02)	8.2 (0.04)	13.8 (0.11)
`Boruta`	27.8 (1.81)	76.6 (6.29)	25.4 (3.91)	37.4 (4.51)	58.6 (3.99)
`Vita`	4.8 (0.06)	10.7 (0.06)	9.2 (0.16)	35.3 (0.72)	89.1 (4.59)
`VSURF`	78.1 (4.14)	190.9 (8.93)	77.0 (28.08)	—	—

Table S4. Average runtimes (in seconds) over 100 trials for each classification experiment. MG stands for Multivariate Gaussian. Standard deviations are in parentheses. Recall that $p = 500$ in the MG experiments and $n = 500$ in the RNA experiments. `VSURF` was too computationally expensive to include when $p = 2000$ and 5000.

S3.2. RNA-seq simulation details. Algorithm S2 describes the data generating procedure for the RNA-seq simulation studies (Section 3.3), which is also depicted in Figure S3. In all steps, "randomly select" means select a parameter uniformly at random from its domain, which are shown in Table S5. The randomized function $f_\theta : \mathbb{R} \to [-1, 1]$ that links the features to the response is

$$f_\theta(x) = \begin{cases} \frac{\delta_1}{2}\left(1 + \tanh(\alpha(\delta_2 x - \beta))\right) & \text{with probability } 1/2, \\ \delta_1 \exp(-\gamma x^2) & \text{with probability } 1/2, \end{cases} \tag{S3.1}$$

where each component of $\theta = (\alpha, \beta, \gamma, \delta_1, \delta_2)$ is drawn uniformly at random prior to each trial according to Table S5. The values of α and γ determine steepness of the curves, β shifts tanh horizontally, and δ_1 and δ_2 reflect the functions about the horizontal and vertical axes, respectively. Figure S4 shows five realizations of f_θ, illustrating the many ways it can influence the response, Y. For example, if the function in the left-most panel of Figure S4 is applied to the genes in a given partition of S, then those genes will only significantly affect Y if their collective expression level is positive, while collective expression levels less than -1 will have virtually no effect on Y.

**Parameter**	α	β	γ	δ_1	δ_2
**Range**	$(0.5, 1.5)$	$(-1, 1)$	$(1, 3)$	$\{-1, 1\}$	$\{-1, 1\}$

TABLE S5. *Simulation parameters.* Parameters are drawn uniformly at random from their corresponding ranges prior to each simulation trial.

Algorithm S2 Data generation for simulation study (one trial)

Input: RNA-seq data $X_{\text{full}} \in \mathbb{R}^{596 \times 6426}$, number of samples n, number of features p, number of true features $|S|$, signal-to-noise ratio SNR, function parameter domain Θ.

Randomly select n rows and p columns of X_{full}. Denote the resulting matrix by $X \in \mathbb{R}^{n \times p}$.
Standardize the columns of X to have mean 0 and variance 1.
Randomly select $|S|$ true features $S \subseteq \{1, \ldots, p\}$.
Randomly select $G \in \{\lfloor |S|/2 \rfloor, \ldots, |S|\}$. Partition S into G disjoint groups, $S = \bigsqcup_{g=1}^{G} S_g$.
Initialize the signal, $\eta \leftarrow (0, \ldots, 0)^{\mathsf{T}} \in \mathbb{R}^n$.
for $g = 1, \ldots, G$ **do**
    $\xi_g \leftarrow \sum_{j \in S_g} X_j$ where $X_j \in \mathbb{R}^n$ is the jth column of X.
    Standardize ξ_g to have mean 0 and variance 1.
    Randomly select a function parameter $\theta \in \Theta$.
    $\eta \leftarrow \eta + f_\theta(\xi_g)$ with f_θ applied to $\xi_g \in \mathbb{R}^n$ componentwise.
end for
For regression: Draw $\epsilon_i \sim \mathcal{N}(0, \sigma^2)$ with $\sigma^2 = \sum_{i=1}^{n} \eta_i^2/(n\,\text{SNR})$ and set $y_i \leftarrow \eta_i + \epsilon_i$.
For classification: Draw $u \sim \text{Uniform}(1, 3)$, then $y_i \sim \text{Bernoulli}(\pi_i)$ where $\pi_i = 1/(1+\exp(-u\eta_i))$.

Output: Features $X \in \mathbb{R}^{n \times p}$, responses $y \in \mathbb{R}^n$, and important features $S \subseteq \{1, \ldots, p\}$.

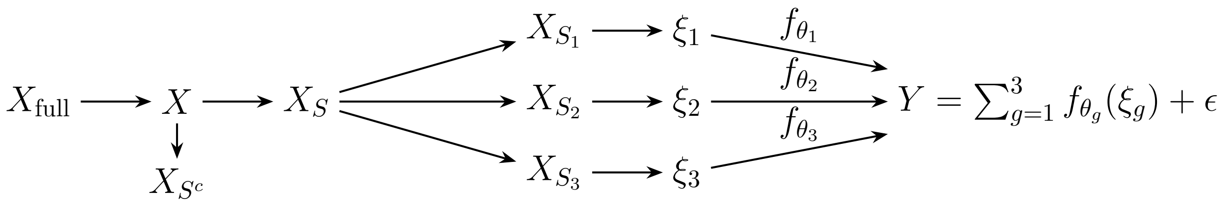

FIGURE S3. *Simulation diagram.* Rows and columns are randomly selected from the full RNA-seq dataset to create a matrix X whose columns are split into important features, X_S, and unimportant features, X_{S^c}. The columns of X_S are further partitioned into G groups; the above figure shows $G = 3$. The features in each group are summed to obtain ξ_g, and a different realization f_{θ_g} of f_θ is applied to ξ_g for each g. For regression, response is the sum of the group-specific signals, $f_{\theta_g}(\xi_g)$, plus noise.

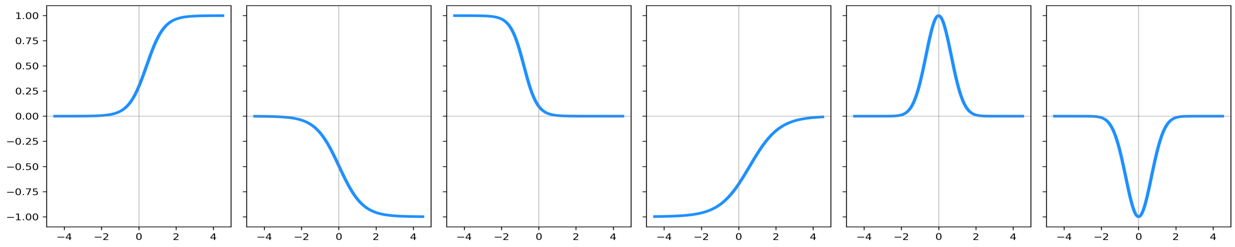

FIGURE S4. *Some realizations of the randomized function f_θ.*

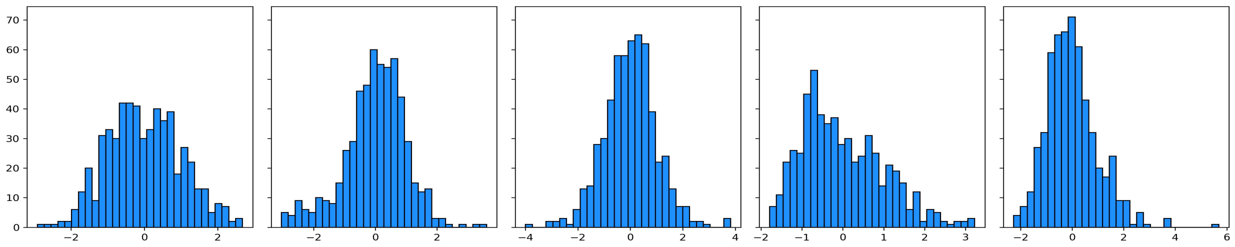

FIGURE S5. *Distributions of five randomly selected genes from the RNA-seq dataset.* Features in the ovarian cancer RNA-seq dataset follow a variety of empirical marginal distributions. For example, the standardized empirical distributions of the five randomly selected genes above, from left to right, are relatively flat, skewed left, approximately Gaussian, skewed right, and contain outliers. Furthermore, the genes exhibit complex correlation structures, with maximum and average absolute pairwise correlations of approximately 0.95 and 0.17 after standardization, respectively.

S4. Cancer study results

We describe our ovarian cancer and glioma studies (Sections S4.1 and S4.2) and present our literature search and cross-validation results (Sections S4.3 and S4.4).

S4.1. Ovarian cancer. For the ovarian cancer cohort, we study the following feature and response combinations (the number of samples and features are shown in parentheses): (i) miRNAs and prognosis ($n = 442$, $p = 585$), (ii) miRNAs and tumor purity ($n = 451$, $p = 585$), (iii) miRNAs and miR-150 ($n = 453$, $p = 584$), (iv) genes and prognosis ($n = 549$, $p = 6426$), and (v) genes and AKT2 ($n = 569$, $p = 6425$). In all cases, missing values were removed. Gene expression levels are measured by RNA-seq. We chose miR-150 and the gene AKT2 as responses in studies (iii) and (v), respectively, because literature searches indicated that both are highly related to ovarian cancer. Literature search results are only reported for clinical responses (prognosis and tumor purity).

S4.2. Glioma. For the glioma cohort, we study the following feature and response combinations (the number of samples and features in each dataset are shown in parentheses): (i) miRNAs and prognosis ($n = 477$, $p = 787$), (ii) miRNAs and miR-155 ($n = 512$, $p = 786$), (iii) genes and prognosis ($n = 625$, $p = 10,058$), and (iv) genes and FOXM1 ($n = 669$, $p = 10,057$). In all cases, missing values were removed. Gene expression levels are measured by RNA-seq. The original glioma RNA-seq dataset contains over $20,000$ genes; we only study the roughly $10,000$ genes in the top 50th percentile of average gene expression (discarding lowly expressed genes is common practice). We chose miR-155 and the gene FOXM1 as responses in studies (ii) and (iv), respectively, because literature searches indicated that both are highly related to glioma. Literature search results are only reported when the response is prognosis.

S4.3. Literature search. In each of the forthcoming studies, we perform literature searches to validate our findings. For the microRNA (miRNA) and ovarian cancer prognosis study, we briefly summarize literature supporting each miRNA selected by at least one feature selection method. Providing such summaries for every study is beyond the scope of this work. Thus, to roughly quantify the relevance of selected features in subsequent studies, we searched the feature name, cancer type, and the word "prognosis" in Europe PMC, an open-access database containing millions of life sciences publications (Europe PMC Consortium, 2015). We then report the total number of citations among all articles returned by the search. For example, in Table S6, searching "miR-93" + "ovarian cancer" + "prognosis" in Europe PMC returned 43,996 citations. For each method, we also include the number of features it selected that had above a certain number of citations, below a certain number of citations, and the total number of features it selected below a certain target FDR. We emphasize that these metrics are less important than the papers themselves. Citation counts favor older publications, and our search criterion does not guarantee relevance to the specific problem at hand (though the summaries below suggest that at least some results are meaningful).

Below, we briefly summarize literature relating miRNAs in Table S6 to ovarian cancer. Additional details are available in the associated references.

miR-1-2. Kandettu et al. (2022) found that miR-1-2 is differentially expressed between cancerous and non-cancerous ovarian cancer cells, but we found no literature linking miR-1-2 to prognosis.

miR-30d. Ye et al. (2015) found that miR-30d suppresses ovarian cancer progression by reducing the levels of Snail, a protein involved in making cancer cells more invasive. They concluded that

miR-30d could be used as a treatment for ovarian cancer. Lee et al. (2012) found that miR-30d is associated with "significantly better disease-free or overall survival" in ovarian cancer patients.

miR-93. Fu et al. (2012) found that miR-93 is significantly upregulated in ovarian cancer cells that are resistant to the chemotherapy drug cisplatin. They also found that miR-93 targets the tumor suppressor gene PTEN and plays a role in the AKT signaling pathway. They concluded that further study of miR-96 may yield therapeutic strategies for overcoming cisplatin-resistant ovarian cancer cells. Meng et al. (2015) found that miR-93 is a potential biomarker of ovarian cancer.

miR-96. Liu et al. (2019) found that overexpression of miR-96 promotes cell proliferation and migration in ovarian cancer cells. They conclude that targeting miR-96 is a potentially promising strategy for treating ovarian cancer. They also report that miR-96 inhibits phosphorylation of AKT, a gene identified by IPSSGB as being relevant to ovarian cancer prognosis. Yang et al. (2020) found that individuals with low-levels of miR-96 "suffered more advanced tumor staging and a worse overall survival" and also identified miR-96 as a potential therapeutic target.

miR-150. Jin et al. (2014) found significant associations between miR-150 downregulation and "aggressive clinicopathological features" in ovarian cancer patients, as well as reduced overall and progression-free survival. They also identified miR-150 expression as a prognostic biomarker in ovarian cancer. Kim et al. (2017) found that downregulation of miR-150 is associated with resistance to paclitaxel, a chemotherapy drug used to treat ovarian cancer. They also report that treatment with pre-miR-150 resensitized cancer cells to paclitaxel, making the drug more effective.

miR-342. Dou et al. (2020) found that miR-342 inhibits the proliferation, invasion, and migration of ovarian cancer cells, and promotes the death of these cells. The study also showed that miR-342 decreases the expression of key proteins involved in the Wnt/β-catenin signaling pathway, which may explain its effects on reducing ovarian cancer cell viability and growth.

miR-1270. Ghafouri-Fard et al. (2022) found that miR-1270 plays a role in sensitivity to the chemotherapy drug cisplatin.

miR-1301. Yu and Gao (2020) found that targeting miR-1301 can inhibit the proliferation of cells that are resistant to the chemotherapy drug cisplatin, thus reducing the occurrence and development of drug-resistant ovarian cancer.

**miRNA**	**Citations**	IPSSGB	IPSSRF	IPSSL1	KOGLM	KORF	KOL1	DeepPINK	SSBoost
miR-93	43996	0.35	0.11	–	–	–	–	–	–
miR-148a	42177	–	0.39	–	–	–	–	–	–
miR-150	41195	0.23	0.39	–	–	–	–	–	–
miR-96	23010	0.23	–	0.47	–	–	–	–	–
miR-342	20291	–	0.39	0.23	–	–	–	–	–
miR-30d	19267	0.23	0.33	–	–	–	–	–	–
miR-301b	3224	0.35	–	–	–	–	–	–	–
miR-1270	2543	0.28	0.11	0.21	–	–	–	–	–
miR-1301	1390	0.35	–	–	–	–	–	–	–
miR-1-2	1220	0.35	–	0.21	–	–	–	–	–
≥ 1000	–	8	6	4	0	0	0	0	0
< 1000	–	0	0	0	0	0	0	0	0
Total	–	8	6	4	0	0	0	0	0

TABLE S6. *MicroRNAs and prognosis (ovarian cancer).* MiRNAs are ordered by citation count. A missing q-value indicates the miRNA was assigned a q-value of less than 0.5 by the corresponding method. The bottom rows report, for each method, the number of selected features with over 1000 citations, under 1000 citations, and the total number selected at the maximum target FDR of 0.5.

**miRNA**	**Citations**	IPSSGB	IPSSRF	IPSSL1	KOGLM	KORF	KOL1	DeepPINK	SSBoost
miR-21	190920	–	0.08	–	–	–	–	–	–
miR-155	128839	0.32	0.08	0.09	–	–	0.20	–	–
miR-145	89265	–	0.24	–	–	–	–	–	–
miR-146a	57373	–	0.03	–	–	–	–	–	–
miR-214	57253	–	0.05	–	–	–	–	–	–
miR-223	53770	0.24	0.03	0.09	–	–	–	–	–
miR-25	43636	0.12	0.32	0.09	–	–	0.20	–	–
miR-22	42211	0.04	0.03	0.07	–	–	0.20	–	0.17
miR-150	41195	0.04	0.03	0.07	–	–	0.20	–	0.17
miR-142	39980	0.04	0.03	–	–	–	–	–	0.25
miR-335	33200	0.26	–	–	–	–	–	–	–
miR-15b	30988	0.24	–	0.20	–	–	0.25	–	–
miR-140	29667	0.04	0.03	–	–	–	–	–	0.17
miR-152	25767	–	0.16	–	–	–	–	–	–
≥ 1000	–	14	25	12	0	0	7	0	5
< 1000	–	0	3	1	0	0	1	0	0
Total	–	14	28	13	0	0	8	0	5

TABLE S7. *MicroRNAs and tumor purity (ovarian cancer).* MiRNAs are ordered by citation count. A missing q-value indicates the miRNA was assigned a q-value of less than 0.35 by the corresponding method. The bottom rows report, for each method, the number of selected features with over 1000 citations, under 1000 citations, and the total number selected at the maximum target FDR of 0.35.

**Gene**	**Citations**	IPSSGB	IPSSRF	IPSSL1	KOGLM	KORF	KOL1	DeepPINK	SSBoost
CD38	109641	0.13	0.14	–	–	–	–	–	–
AKT2	97889	0.13	–	–	0.30	–	–	–	–
ERBB4	61018	–	–	–	–	–	0.45	–	–
CCR3	25401	–	0.23	–	–	0.35	–	–	–
CD1C	24420	–	0.38	–	–	–	–	–	–
WTAP	22418	0.24	–	–	–	–	–	–	–
SHMT2	19844	–	–	–	0.30	–	–	–	–
AAAS	16226	–	–	–	0.30	–	0.45	–	–
PAK4	14396	–	0.38	–	–	–	–	–	–
SLAMF7	12027	0.24	0.14	–	–	–	–	–	–
≥ 200	–	15	14	8	13	2	7	0	0
< 200	–	4	1	3	4	1	0	0	0
Total	–	19	15	11	17	3	7	0	0

Table S8. *RNA-seq and prognosis (ovarian cancer).* Genes are ordered by citation count. A missing q-value indicates the gene was assigned a q-value of less than 0.5 by the corresponding method. The bottom rows report, for each method, the number of selected features with over 200 citations, under 200 citations, and the total number selected at the maximum target FDR of 0.5.

**miRNA**	**Citations**	IPSSGB	IPSSRF	IPSSL1	KOGLM	KORF	KOL1	DeepPINK	SSBoost
miR-155	94172	–	0.05	–	–	–	–	–	–
miR-10b	42000	0.21	0.05	0.03	–	–	0.35	–	0.39
miR-148a	32559	–	0.13	–	–	–	–	–	–
miR-335	22423	–	0.08	0.05	–	–	0.50	–	–
miR-15b	21960	0.14	0.05	0.03	–	–	0.20	–	0.38
miR-424	21680	–	0.37	–	–	–	–	–	–
miR-10a	20065	–	0.21	–	–	–	–	–	–
miR-224	18932	0.16	–	–	–	–	–	–	0.38
miR-503	11894	0.14	0.06	–	–	–	–	–	0.38
let-7e	11348	0.25	0.06	–	–	–	–	–	0.38
≥ 100	–	15	21	6	0	0	14	0	14
< 100	–	5	2	3	0	0	10	0	4
Total	–	20	23	9	0	0	24	0	18

Table S9. *MicroRNAs and prognosis (glioma).* MiRNAs are ordered by citation count. A missing q-value indicates the miRNA was assigned a q-value of less than 0.5 by the corresponding method. The bottom rows report, for each method, the number of selected features with over 100 citations, under 100 citations, and the total number selected at the maximum target FDR of 0.5.

Gene	Citations	IPSSGB	IPSSRF	IPSSL1	KOGLM	KORF	KOL1	DeepPINK	SSBoost
FOXM1	62538	0.25	–	–	–	–	–	–	–
WEE1	26648	0.10	0.06	–	–	0.44	–	–	–
IGFBP2	24482	–	0.08	–	–	–	–	–	–
CX3CL1	23044	–	–	–	–	–	0.34	–	–
TIMP1	22632	–	–	–	–	0.44	–	–	–
SKI	19220	0.12	0.23	0.03	–	0.44	0.34	–	–
CCNB1	14856	–	0.10	–	–	–	–	–	–
CDK9	14558	0.25	–	–	–	–	–	–	–
TOP2A	13820	–	–	0.03	–	–	–	–	–
PDPN	12929	–	0.23	–	–	–	–	–	–
MSN	11866	–	0.10	–	–	–	–	–	–
ATF2	11040	0.10	–	–	–	–	–	–	–
≥ 500	–	19	18	12	0	22	24	0	0
< 500	–	9	6	7	0	15	36	0	0
Total	–	28	24	19	0	37	60	0	0

TABLE S10. *RNA-seq and prognosis (glioma).* Genes are ordered by citation count. A missing q-value indicates the gene was assigned a q-value of less than 0.5 by the corresponding method. The bottom rows report, for each method, the number of selected features with over 500 citations, under 500 citations, and the total number selected at the maximum target FDR of 0.5.

S4.4. Cross-validation. As noted in the main text, we also measure feature selection performance by implementing a 20-fold cross-validation (CV) procedure, described as follows. In each of the 20 CV steps, one group of patients is set aside (the test set), and a set of features is selected by each method using the data in the remaining groups (the training set). Next, for each method, we construct three predictive models—a linear model, a random forest model, and a gradient boosting model—using only the features selected by that method on the training data. Each model is then used to predict responses from the test set, and the smallest of the three prediction errors is recorded (we use mean squared error for regression and 1 − accuracy for classification). All three models are implemented to ensure that no method has an inherent advantage over another. For example, the features selected by `IPSSL1` may be better suited to minimizing error in a linear model than those selected by `IPSSGB`, while those selected by `IPSSGB` may be better suited to minimizing error in a gradient boosting model than the ones selected by `IPSSL1`. The linear and random forest predictive models are implemented with scikit-learn (Pedregosa et al., 2011) and gradient boosting with `XGBoost` (Chen and Guestrin, 2016), always with default parameters. For continuous responses, in each CV step we subtract the mean of the training responses from all responses, training and test, and scale all responses by the empirical standard deviation of the training responses.

CV study results are shown in Figures S6–S11. Each plot contains two subplots. The left subplots show the prediction error associated to the features selected by each method at the given target FDR. In all studies, we find that the three IPSS methods select features at much lower target FDRs than all of the model-X knockoffs methods. The right subplots show the prediction error as a function of the number of features selected by each method. Curves for each method in these plots are obtained by varying the target FDR between 0 and 0.5. `Boruta` does not have FDR control parameters and is therefore represented by a single point in these plots. In each plot, the dashed black line shows

the average error when using all features in the dataset to predict the response variable. `DeepPINK` rarely selects any features and is therefore represented by a single point for better visibility.

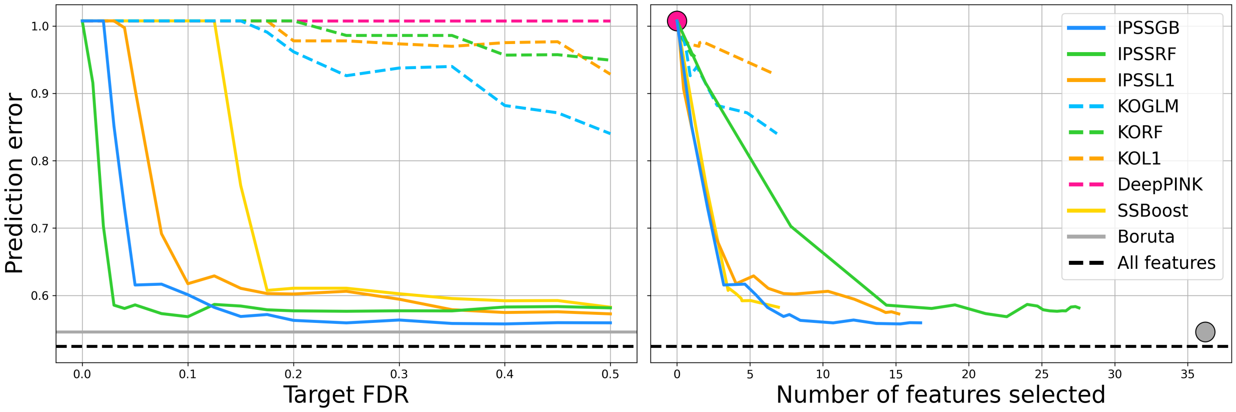

FIGURE S6. *MiRNAs and tumor purity (ovarian cancer).*

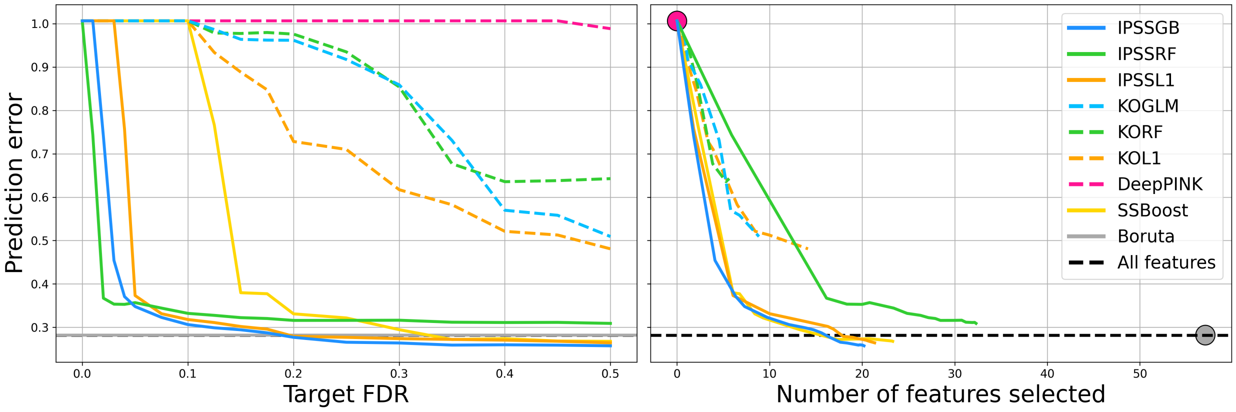

FIGURE S7. *MiRNAs and miR-150 (ovarian cancer).*

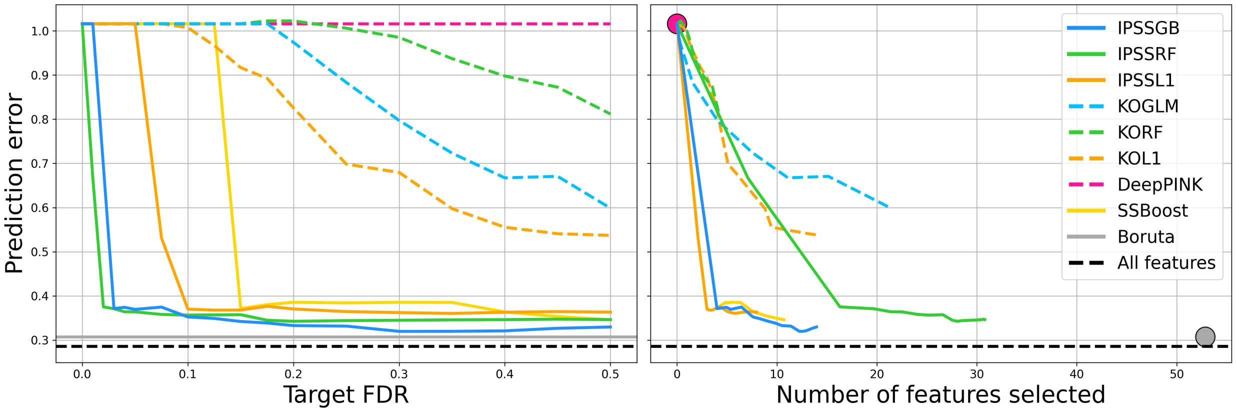

FIGURE S8. *Genes and AKT2 (ovarian cancer).*

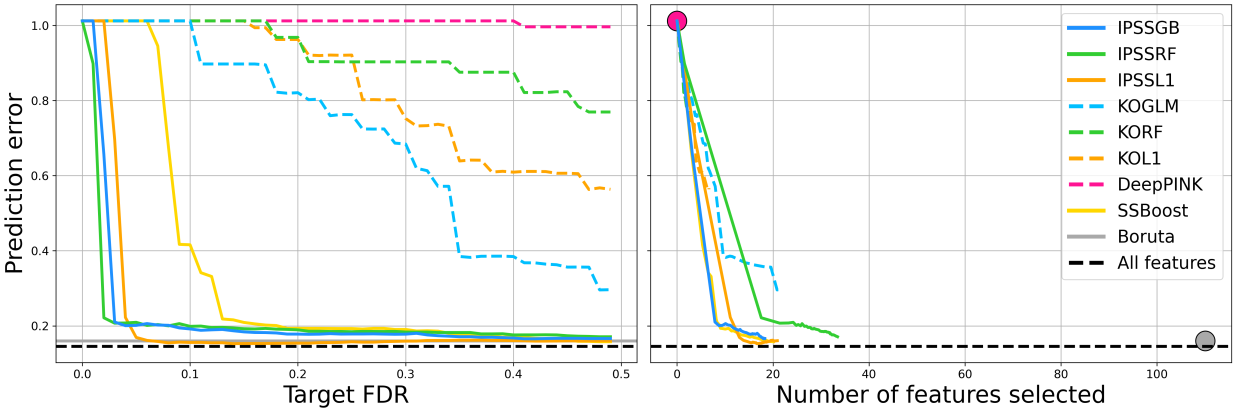

Figure S9. *MiRNAs and miR-155 (glioma).*

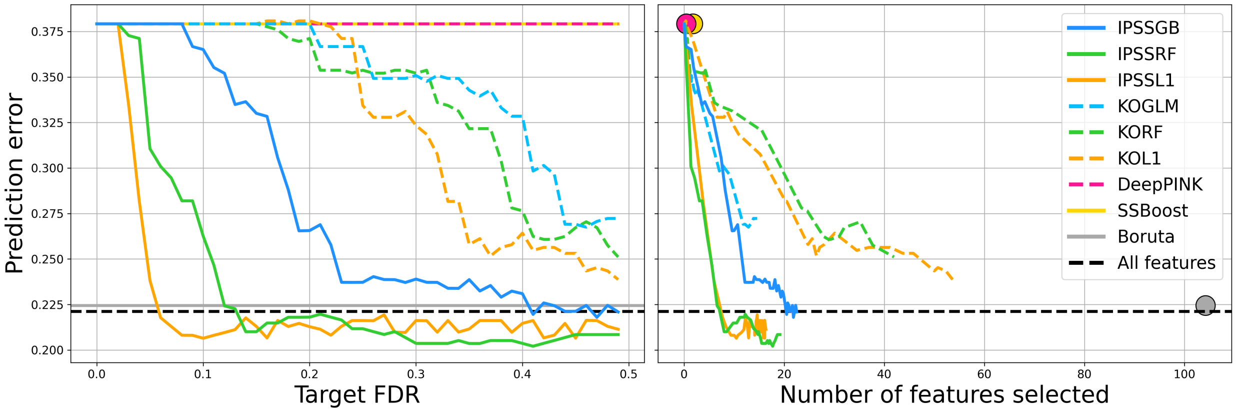

Figure S10. *Genes and prognosis (glioma).*

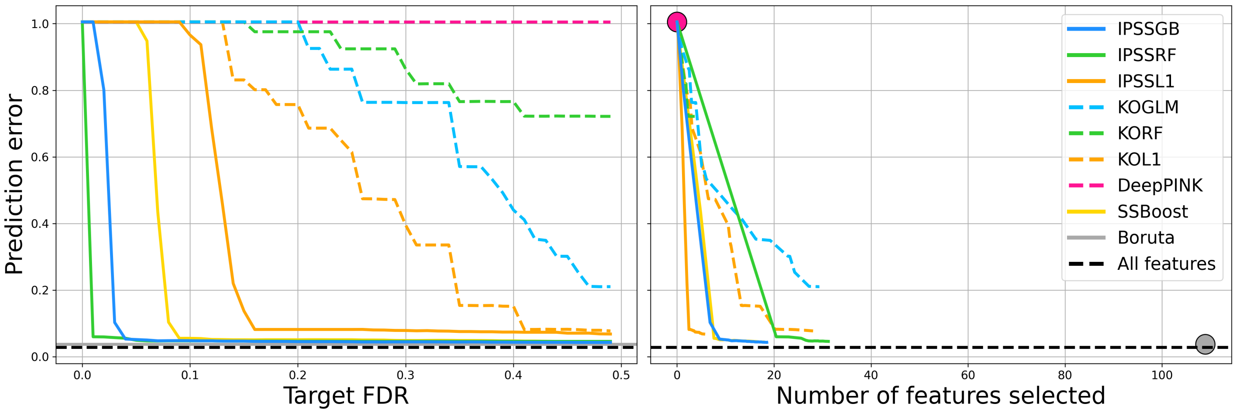

Figure S11. *Genes and FOXM1 (glioma).*

Figures S12–S19 show the sensitivity of `IPSSGB` to the IPSS parameters discussed in Sections S1.3 and 2. Data are simulated according to the ovarian cancer RNA-seq simulation design described in Sections S3.2 and 3.3 for both regression and classification. The results for `IPSSRF` were similar and are therefore omitted.

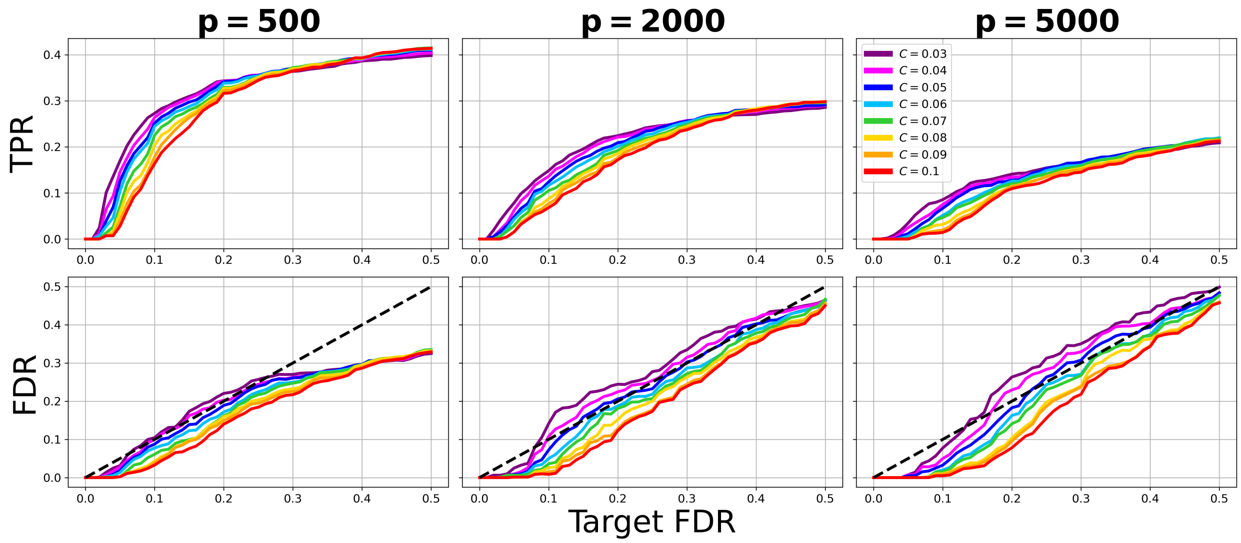

Figure S12. *Different choices of C (regression).*

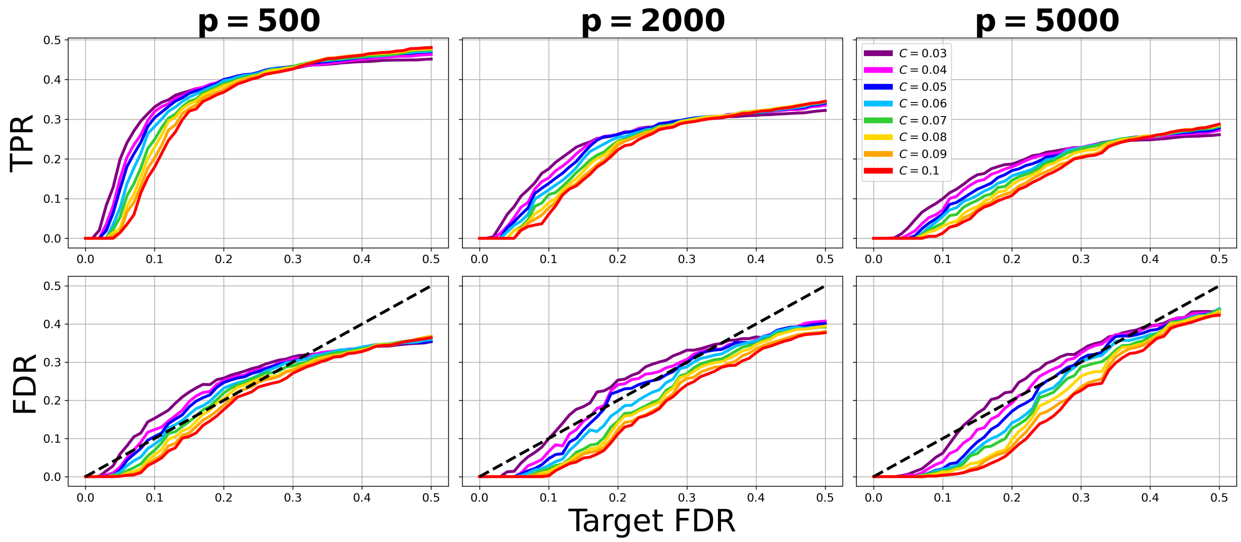

Figure S13. *Different choices of C (classification).*

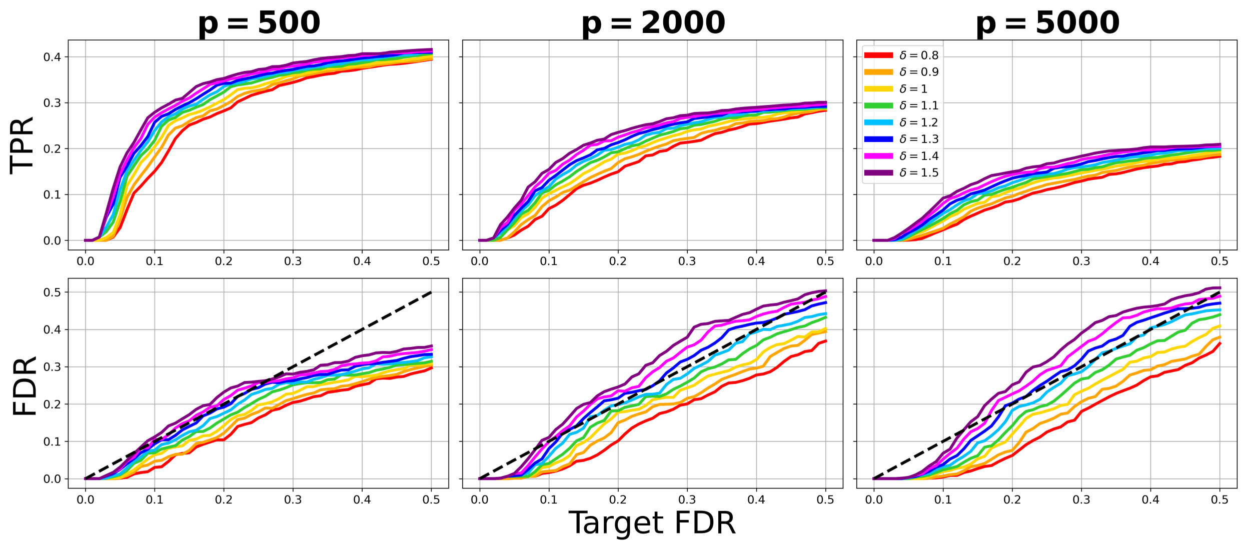

FIGURE S14. *Different choices of δ (regression).*

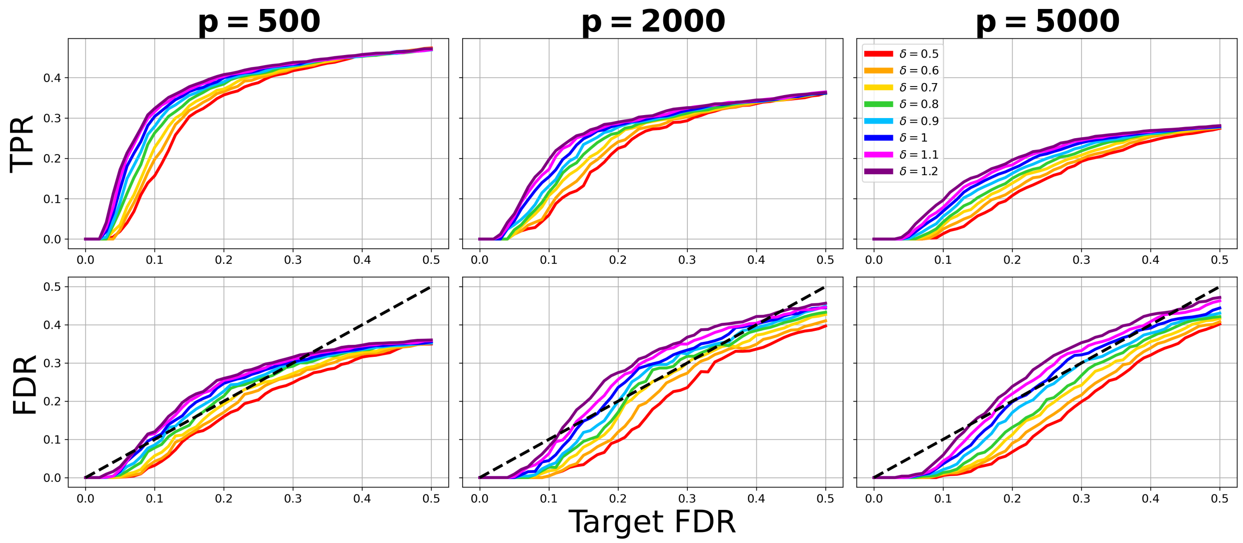

FIGURE S15. *Different choices of δ (classification).*

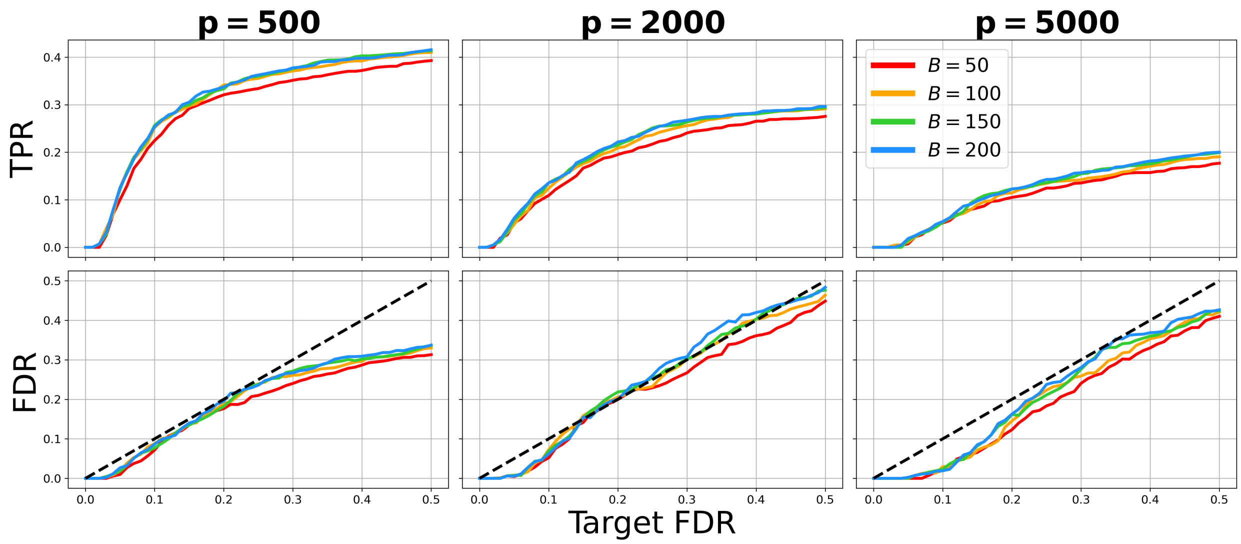

FIGURE S16. *Different choices of B (regression).*

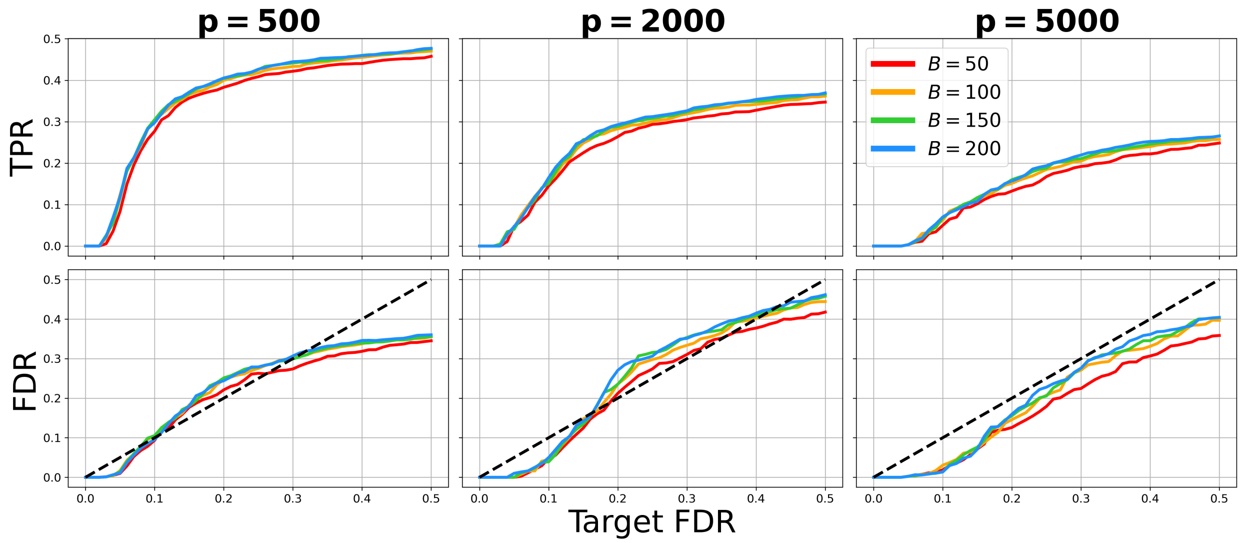

FIGURE S17. *Different choices of B (classification).*

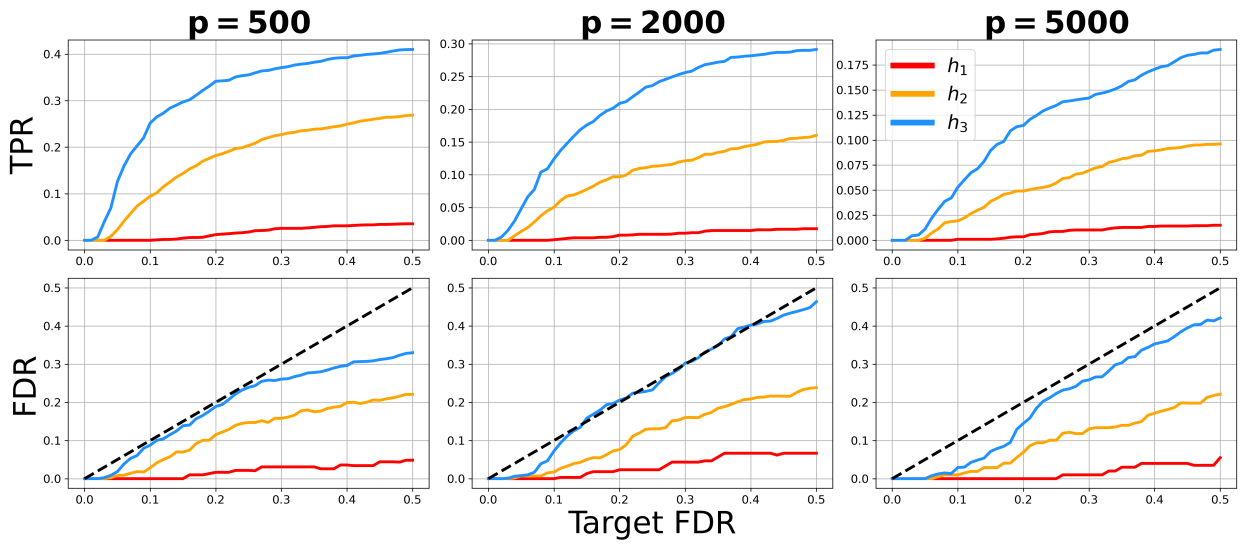

FIGURE S18. *Different choices of function (regression).*

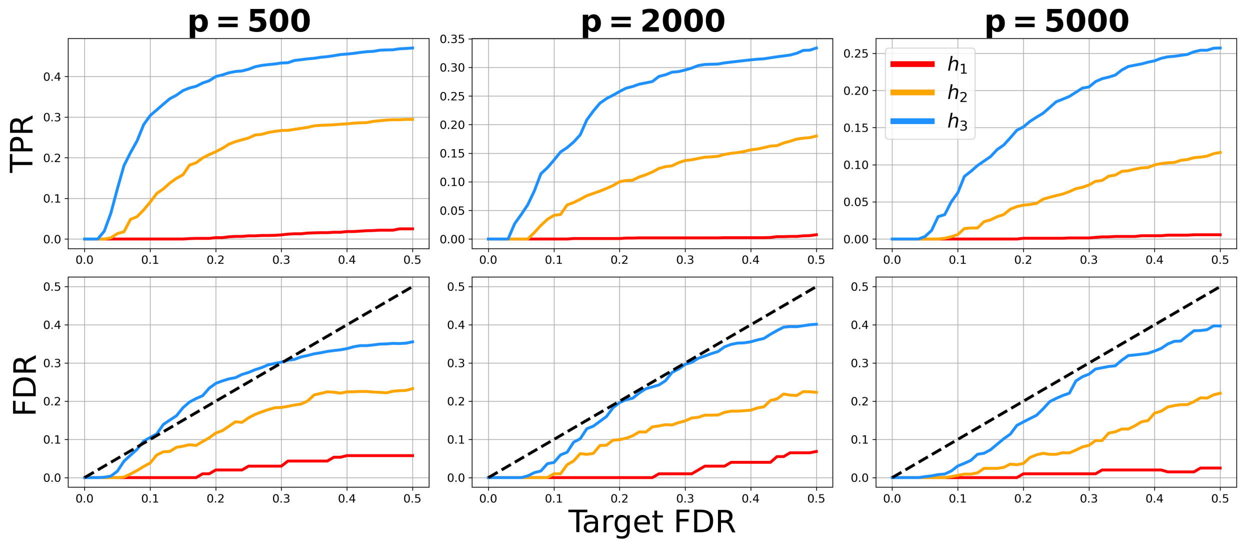

FIGURE S19. *Different choices of function (classification).*

E. Candes, Y. Fan, L. Janson, and J. Lv. Panning for gold: 'model-x' knockoffs for high dimensional controlled variable selection. *Journal of the Royal Statistical Society Series B: Statistical Methodology*, 80(3):551–577, 2018.

T. Chen and C. Guestrin. Xgboost: A scalable tree boosting system. In *Proceedings of the 22nd ACM SIGKDD International Conference on Knowledge Discovery and Data Mining*, pages 785–794, 2016.

T. Coleman, W. Peng, and L. Mentch. Scalable and efficient hypothesis testing with random forests. *Journal of Machine Learning Research*, 23(170):1–35, 2022.

Y. Dou, F. Chen, Y. Lu, H. Qiu, and H. Zhang. Effects of Wnt/β-catenin signal pathway regulated by miR-342-5p targeting CBX2 on proliferation, metastasis and invasion of ovarian cancer cells. *Cancer Management and Research*, pages 3783–3794, 2020.

Europe PMC Consortium. Europe PMC: a full-text literature database for the life sciences and platform for innovation. *Nucleic Acids Research*, 43(D1):D1042–D1048, 2015.

J. H. Friedman, T. Hastie, and R. Tibshirani. Regularization paths for generalized linear models via coordinate descent. *Journal of Statistical Software*, 33:1–22, 2010.

X. Fu, J. Tian, L. Zhang, Y. Chen, and Q. Hao. Involvement of microRNA-93, a new regulator of PTEN/Akt signaling pathway, in regulation of chemotherapeutic drug cisplatin chemosensitivity in ovarian cancer cells. *FEBS Letters*, 586(9):1279–1286, 2012.

R. Genuer, J.-M. Poggi, and C. Tuleau-Malot. Variable selection using random forests. *Pattern Recognition Letters*, 31(14):2225–2236, 2010.

S. Ghafouri-Fard, T. Khoshbakht, B. M. Hussen, S. Sarfaraz, M. Taheri, and S. A. Ayatollahi. Circ_CDR1as: A circular RNA with roles in the carcinogenesis. *Pathology-Research and Practice*, 236:153968, 2022.

B. Hofner and T. Hothorn. stabs: Stability selection with error control. *R package version 0.6-3*, 2017.

B. Hofner, A. Mayr, N. Robinzonov, and M. Schmid. Model-based boosting in R: a hands-on tutorial using the R package mboost. *Computational Statistics*, 29:3–35, 2014.

B. Hofner, L. Boccuto, and M. Göker. Controlling false discoveries in high-dimensional situations: boosting with stability selection. *BMC Bioinformatics*, 16:1–17, 2015.

M. Jin, Z. Yang, W. Ye, H. Xu, and X. Hua. MicroRNA-150 predicts a favorable prognosis in patients with epithelial ovarian cancer, and inhibits cell invasion and metastasis by suppressing transcriptional repressor ZEB1. *PloS One*, 9(8):e103965, 2014.

A. Kandettu, D. Adiga, V. Devi, P. S. Suresh, S. Chakrabarty, R. Radhakrishnan, and S. P. Kabekkodu. Deregulated miRNA clusters in ovarian cancer: Imperative implications in personalized medicine. *Genes & Diseases*, 9(6):1443–1465, 2022.

T. H. Kim, J.-Y. Jeong, J.-Y. Park, S.-W. Kim, J. H. Heo, H. Kang, G. Kim, and H. J. An. miR-150 enhances apoptotic and anti-tumor effects of paclitaxel in paclitaxel-resistant ovarian cancer cells by targeting Notch3. *Oncotarget*, 8(42):72788, 2017.

H. Lee, C. S. Park, G. Deftereos, J. Morihara, J. E. Stern, S. E. Hawes, E. Swisher, N. B. Kiviat, and Q. Feng. MicroRNA expression in ovarian carcinoma and its correlation with clinicopathological features. *World Journal of Surgical Oncology*, 10:1–10, 2012.

B. Liu, J. Zhang, and D. Yang. miR-96-5p promotes the proliferation and migration of ovarian cancer cells by suppressing Caveolae1. *Journal of Ovarian Research*, 12:1–9, 2019.

N. Meinshausen and P. Bühlmann. Stability selection. *Journal of the Royal Statistical Society Series B: Statistical Methodology*, 72(4):417–473, 2010.

O. Melikechi and J. W. Miller. Integrated path stability selection. *arXiv preprint arXiv:2403.15877*, 2024.

X. Meng, S. A. Joosse, V. Müller, F. Trillsch, K. Milde-Langosch, S. Mahner, M. Geffken, K. Pantel, and H. Schwarzenbach. Diagnostic and prognostic potential of serum miR-7, miR-16, miR-25, miR-93, miR-182, miR-376a and miR-429 in ovarian cancer patients. *British Journal of Cancer*, 113(9):1358–1366, 2015.

F. Pedregosa, G. Varoquaux, A. Gramfort, V. Michel, B. Thirion, O. Grisel, M. Blondel, P. Prettenhofer, R. Weiss, V. Dubourg, et al. Scikit-learn: Machine learning in python. *Journal of Machine Learning Research*, 12:2825–2830, 2011.

R. D. Shah and R. J. Samworth. Variable selection with error control: another look at stability selection. *Journal of the Royal Statistical Society Series B: Statistical Methodology*, 75(1):55–80, 2013.

R. Tibshirani. Regression shrinkage and selection via the lasso. *Journal of the Royal Statistical Society Series B: Statistical Methodology*, 58(1):267–288, 1996.

N. Yang, Q. Zhang, and X.-J. Bi. miRNA-96 accelerates the malignant progression of ovarian cancer via targeting FOXO3a. *European Review for Medical & Pharmacological Sciences*, 24(1), 2020.

Z. Ye, L. Zhao, J. Li, W. Chen, and X. Li. miR-30d blocked transforming growth factor β1–induced epithelial-mesenchymal transition by targeting snail in ovarian cancer cells. *International Journal of Gynecologic Cancer*, 25(9), 2015.

J.-L. Yu and X. Gao. MicroRNA 1301 inhibits cisplatin resistance in human ovarian cancer cells by regulating EMT and autophagy. *European Review for Medical & Pharmacological Sciences*, 24(4), 2020.
